# High-throughput screens identify HSP90 inhibitors as potent therapeutics that target inter-related growth and survival pathways in advanced prostate cancer

**DOI:** 10.1038/s41598-018-35417-0

**Published:** 2018-11-22

**Authors:** Keith H. Jansson, John B. Tucker, Lauren E. Stahl, John K. Simmons, Caitlyn Fuller, Michael L. Beshiri, Supreet Agarwal, Lei Fang, Paul G. Hynes, Aian Neil Alilin, Ross Lake, Yasmine C. Abbey, Jacob Cawley, Caitlin M. Tice, JuanJuan Yin, Crystal McKnight, Carleen Klummp-Thomas, Xiaohu Zhang, Rajarshi Guha, Shelley Hoover, R. Mark Simpson, Holly M. Nguyen, Eva Corey, Craig J. Thomas, David A. Proia, Kathleen Kelly

**Affiliations:** 1 Laboratory of Genitourinary Cancer Pathogenesis, CCR, National Cancer Institute, Bethesda, MD USA; 2 Laboratory of Cancer Biology and Genetics, CCR, National Cancer Institute, Bethesda, MD USA; 3 Division of Pre-clinical Innovation, NCATS, Rockville, MD USA; 4 Department of Urology, University of Washington, Seattle, WA USA; 5 C4 Therapeutics, Cambridge, MA USA

## Abstract

The development of new treatments for castrate resistant prostate cancer (CRPC) must address such challenges as intrinsic tumor heterogeneity and phenotypic plasticity. Combined *PTEN/*TP53 alterations represent a major genotype of CRPC (25–30%) and are associated with poor outcomes. Using tumor-derived, castration-resistant *Pten/Tp53* null luminal prostate cells for comprehensive, high-throughput, mechanism-based screening, we identified several vulnerabilities among >1900 compounds, including inhibitors of: PI3K/AKT/mTOR, the proteasome, the cell cycle, heat shock proteins, DNA repair, NFκB, MAPK, and epigenetic modifiers. HSP90 inhibitors were one of the most active compound classes in the screen and have clinical potential for use in drug combinations to enhance efficacy and delay the development of resistance. To inform future design of rational drug combinations, we tested ganetespib, a potent second-generation HSP90 inhibitor, as a single agent in multiple CRPC genotypes and phenotypes. Ganetespib decreased growth of endogenous *Pten/Tp53* null tumors, confirming therapeutic activity *in situ*. Fifteen human CRPC LuCaP PDX-derived organoid models were assayed for responses to 110 drugs, and HSP90 inhibitors (ganetespib and onalespib) were among the select group of drugs (<10%) that demonstrated broad activity (>75% of models) at high potency (IC50 <1 µM). Ganetespib inhibits multiple targets, including AR and PI3K pathways, which regulate mutually compensatory growth and survival signals in some forms of CRPC. Combined with castration, ganetespib displayed deeper PDX tumor regressions and delayed castration resistance relative to either monotherapy. In all, comprehensive data from near-patient models presents novel contexts for HSP90 inhibition in multiple CRPC genotypes and phenotypes, expands upon HSP90 inhibitors as simultaneous inhibitors of oncogenic signaling and resistance mechanisms, and suggests utility for combined HSP90/AR inhibition in CRPC.

## Introduction

Prostate cancer is one of the most commonly diagnosed cancers among men in industrialized nations and is a leading cause of cancer-related deaths. Because androgen receptor (AR) signaling is required for the growth of almost all prostate cancers, locally advanced and metastatic disease are treated with androgen deprivation therapy (ADT)^1,2^. Although ADT is initially effective, most men relapse with castrate resistant prostate cancer (CRPC), which usually arises as a result of acquired resistance due to direct or indirect mechanisms that restore AR signaling^1,2^. In addition, it is becoming increasingly appreciated that some men relapse with a clinically aggressive variant which demonstrates reduced AR signaling requirements and displays differentiation plasticity, often associated with markers of neuroendocrine differentiation^3^. There are limited therapies approved for CRPC; the most common involve targeting reactivated AR signaling (enzalutamide, abiraterone)^1,2^ or microtubule inhibitors (cabazitaxel, docetaxel)^4^. More recently, treatment of *BRCA*1*/2* mutant CRPC with the PARP inhibitor, olaparib^5^, has received breakthrough designation by the FDA. Generally, the efficacy of second-line CRPC therapies is short-lived, extending survival by <6 months.

Histological and genomic characterization of metastatic CRPC (mCRPC) have revealed significant intrapatient and interpatient heterogeneity, suggesting that genomic instability coupled with multiclonal selection can mechanistically account for a significant fraction of resistance^6^. Because several mechanisms of potential resistance may be evolving concurrently in any individual patient, a promising approach concerns addressing heterogeneous resistance pathways early in treatment with drug combinations and/or individual drugs that simultaneously inactivate multiple relevant targets.

AR is the most frequently altered gene in CRPC, consistent with acquired resistance to ADT. The second most frequent genomic alterations in CRPC occur in *TP53*^7^, observed in about 50% of clinical samples. *TP53* alterations appear to be an important determinant of metastatic potential^8^. *PTEN* is the third most altered locus, occurring in approximately 40% of mCRPC^7^. *PTEN* loss predicts increased future risk of biochemical recurrence in patients undergoing prostatectomy, decreased time to metastasis, increased PrCa specific mortality in high-risk cohorts^9–11^ and promotes acquisition of castration resistance in mouse models^12^. Combined mutations in *PTEN* and *TP53* occur early^13^, represent a major genotype of CRPC (observed in 20–30% of tumors)^7^, and are associated with a poor clinical prognosis^14^. *Pten/Tp53* null mouse models of PrCa have revealed differentiation plasticity as a mechanistic factor of disease aggressiveness, observed as the amplification of an immature, androgen independent luminal stem/progenitor population^15^ as well as a relatively high potential to evolve abiraterone-selected populations of neuroendocrine PrCa^16^. A high-throughput screen of ~2000 mechanistically defined compounds against multiple tumor-derived, *Pten/Tp53* null PrCa cell lines identified HSP90 inhibitors as one of the most potent anti-proliferative classes of compounds.

HSP90 is an ATP-dependent molecular chaperone that is part of a multi-chaperone complex, which regulates the activation and stability of a diverse array of >200 client proteins^17^. Cancer cells depend upon HSP90 to maintain oncoprotein activity, to buffer cellular stresses that are increased in the malignant state^18^, and to stabilize evolving resistance traits^19^. HSP90 inhibitors have demonstrated activity in small numbers of AR-dependent and independent CRPC cell lines^20,21^, stimulating interest in clinical investigations as a single agent and in combination with other compounds. However, PrCa cell lines do not accurately reflect the genomic landscape of clinical CRPC leading to questions about translational accuracy in patients. PDXs recapitulate many of the clinically important features of natural tumors such as cellular heterogeneity, and they have been shown to be predictive of genomically-matched patient responses to drug treatments^22^. Recent advances for culturing CRPC PDX-derived organoids *ex vivo*^23^ have afforded a unique opportunity to analyze the genomic and histological specificity of second generation HSP90 inhibitor responses. To address the context for HSP90 inhibitor efficacy in CRPC, we assayed the activity of ganetespib in an intrinsically castration resistance *Pten/Tp53* null mouse model of PrCa and in PDX models that reflect clinical CRPC genotypic and phenotypic heterogeneity. We found that HSP90 inhibitors are potent and broadly active across multiple CRPC models. Due to their extensive target specificity and ability to stabilize acquired genetic variations, HSP90 inhibitors may be particularly useful in combination with cytotoxic or targeted therapies to combat rapidly evolving resistance mechanisms. As AR-directed therapies represent first and usually second line treatment for metastatic PrCa^1,2^, we evaluated the effect of ganetespib in combination with ADT. We present pre-clinical *in vivo* data demonstrating the efficacy of ganetespib for delaying castration resistance in *PTEN/TP53* altered CRPC.

## Results

### High-throughput MIPE screen elucidates sensitivities of *Pten*/*Tp53* null PrCa cells to targeted therapies

**Figure 1:**
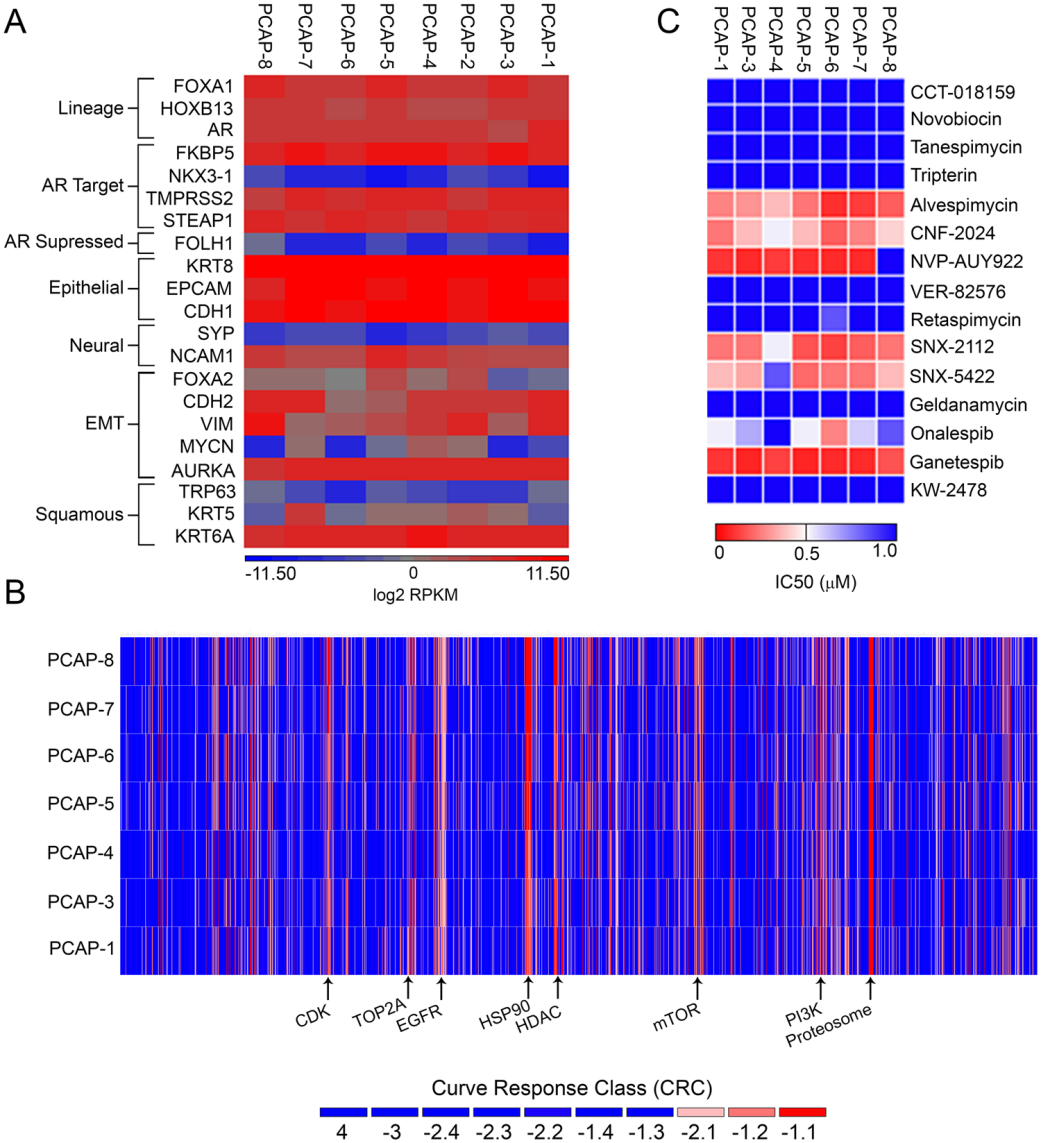
High-throughput MIPE screen identifies specific classes of compounds, including potent HSP90 inhibitors. (**A**) RNAseq intensity plot of select PrCa genes. (**B**) Heat map of curve response class activity from MIPE screen. (**C**) Heat map of MIPE screen HSP90 inhibitor IC50 values.

Non-biased, comprehensive pharmacogenomics has been relatively under utilized in PrCa research due to a lack of amenable models. To address the common and aggressive CRPC genotype of combined *PTEN/TP53* aberrations, we have applied a high-throughput screen to cell lines derived from the *PBCre-4;Pten*^*fl/fl*^,*Tp53*^*fl/fl*^ model, which contain a dominant castration-resistant population with features of plasticity^24^. We derived cell lines from tumors obtained from intact mice or mice previously treated with degarelix (DGX), a form of chemical castration used in patients. To account for the development of non-uniform secondary mutations in tumors growing in androgen intact or depleted conditions, we derived eight *Pten*/*Tp53* null cell lines from independent tumors harvested from four non-treated (PCAP-1–4) and four degarelix-treated (PCAP-5–8) mice (Supplementary Fig. 1A). RNAseq (Fig. 1A) and protein marker analyses characterized the lines as luminal epithelium (KRT8^+^/AR^+^/CK5^low^) expressing plasticity/EMT (CDH2, VIM) markers. The lines were indistinguishable with respect to treatment history (Fig. 1A, Supplementary Fig. 1B), consistent with *Pten/Tp53* loss rendering a large population of cells androgen indifferent^12^. The presence of androgen in the growth media did not appear to have a substantial effect as PCAP-4, which was phenotypically similar to the other lines, was derived and maintained in the presence of DHT. We used seven of these cell lines to identify pathways of therapeutic vulnerabilities by screening the Mechanism Interrogation PlatE oncology library (MIPE), which consists of 1,912 small molecule compounds with known targets^25^. Post-screen informatics data processing and subsequent analysis sorted out compounds that displayed strong dose-dependent activity (−1.1 and −1.2 curve response class). Several compounds were unique to each cell line, confirming the importance of screening multiple biological replicates. No difference in sensitivity was observed comparing PCAP 1–4 and PCAP 5–8 cell lines (Fig. 1B), suggesting there was no consistent underlying selection for survival in DGX conditions. Grouping PCAP cell lines together as one population identified a diverse array of 230 compounds (12% of MIPE library) with robust activity against at least 4 out of 7 of the cell lines (Fig. 1B, Supplementary Table 3). A majority of the 230 active compounds redundantly targeted components of signaling and regulatory pathways important in PrCa, including: 29 compounds targeting PI3K/AKT/mTOR signaling (AKT1, PIK3CA, mTORC1/2), 8 topoisomerase inhibitors (TOP1, TOP2A), 8 tubulin inhibitors, 7 compounds targeting EGFR/HER2, 7 compounds targeting DNA repair pathways (ATR, CHEK1, PRKDC), 5 compounds targeting NFKB signaling (NFKB1, IKBKB), 6 compounds targeting MAPK pathways (RAF, MEK, ERK,), and 3 epigenetic inhibitors (BRD4, DNMT1) (Supplementary Table 3). Other active compound classes included 15 compounds targeting cell cycle regulatory proteins (CDC25, CDKs, WEE1), 12 HDAC inhibitors, and 9 proteasome inhibitors (Supplementary Table 3). Several compound classes were notably less active, such as: AR signaling inhibitors (17 of 17 compounds), Aurora kinase inhibitors (21 of 21 compounds), PARP inhibitors (8 of 8 compounds), and WNT pathway inhibitors (6 of 7 compounds) (Supplementary Table 4). Patterns of sensitivity and resistance from the MIPE screen are consistent with previous work demonstrating activity for PI3K and TORC1/TORC2 inhibitors for *Pten* deleted cancers^26,27^ and increased resistance to AR antagonists has been described in *Pten*/*Tp53* null mouse models^12,15^. The strong activity of EGFR inhibitors was unusual in the context of PTEN deficiency^28,29^ but some of this activity may be explained by a dependence upon EGF contained in the cell culture medium^30^. HSP90 inhibitors were notable for their overall potency in the screen (Fig. 1B). HSP90 inhibitors are of interest due to 1) the potential of chaperone-dependent protein homeostasis as a cancer vulnerability, 2) the utility of HSP90 inhibition in a heterogeneous disease with several poorly-druggable oncogenic drivers, 3) the reduced toxicity of second-generation HSP90 inhibitors, and 4) the potential of HSP90 inhibitors in drug combinations.

**Figure 2:**
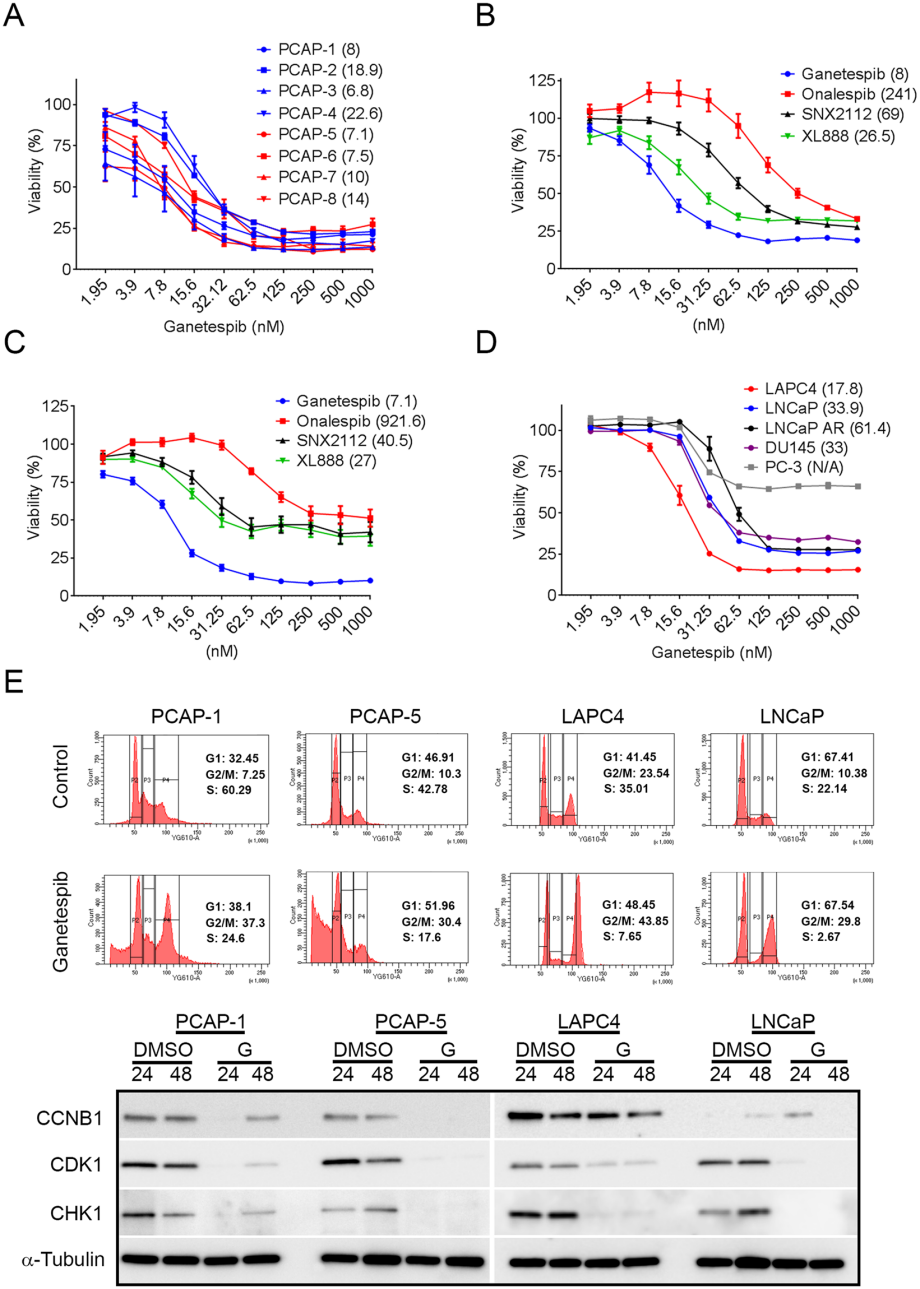
Validation of HSP90 inhibitor activity and ganetespib effects on cell cycle profiles. (**A**) Ganetespib dose response experiments on eight PCAP cell lines. Each point represents the average of three independent experiments (n = 3). (**B**,**C**) Dose response assays comparing ganetespib activity to onalespib, SNX2112, and XL888 in PCAP-1 (**B**, n = 3) and PCAP-5 cell lines (**C**, n = 3). (**D**) Ganetespib dose response on human PrCa cell lines (n = 3). All dose response data presented as percent viability +/− SEM. IC50 values are in parenthesis next to cell or compound name. (**E**) Mouse and human PrCa cell lines were treated with DMSO or 125 nM ganetespib and assayed for changes in cell cycling at 24 hours. Flow cytometry curves are representative of at least 3 separate experiments. PrCa cell lines were exposed to DMSO or 125 nM ganetespib (G) for 24 or 48 hours and probed for G2/M progression/checkpoint proteins. Western blots analyses are representative of three individual experiments. Vertical white space between images indicates separate gels.

HSP90 inhibitors included in the screen demonstrated different degrees of potency, with the second-generation inhibitor, ganetespib^31^, displaying lower IC50 values than all other HSP90 inhibitors in the screen, including other clinically-used resorcinol analogues, onalespib and NVP-AUY922 (Fig. 1C). When the observed response at the maximum concentration (MAXR) was ranked for all 1,912 compounds, ganetespib fell into the most active 20% of all compounds screened for each PCAP cell line. Overall, the PCAP cell line MAXR values for ganetespib were lower than 50% of cell lines previously screened at NCATS (Supplementary Fig. 2A). Ganetespib validated in all mouse *Pten*/*Tp53* null cell lines with sigmoidal dose response curves which exhibited IC50 values ≤23 nM and >95% loss of viability (Fig. 2A). As was suggested by the MIPE screen, ganetespib was the most potent HSP90 inhibitor against select PCAP cell lines (Fig. 2B,C). Indeed, ganetespib induced HSP70 expression at lower concentations compared to onalespib in PCAP cells (Supplementary Fig. 2B), suggesting on-target potency effects. Although several classes of HSP90 inhibitors have a similar mechanism of action^32^, it has been postulated that the exceptional activity and favorable safety of ganetespib are due to the absence of a benzoquinone component, enhanced lipophilic properties, and a smaller molecular weight compared to other HSP90 inhibitors^33^. Also, we characterized the efficacy of ganetespib on a panel of commonly-used, heterogeneous, established human PrCa cell lines. Both the androgen sensitive and androgen insensitive cell lines were inhibited by ganetespib (Fig. 2D). These data validated the potent effect of ganetespib on cell viability and showed that ganetespib efficacy extends to phenotypically and genotypically heterogeneous human PrCa cell lines.

### Ganetespib induces cell cycle arrest in PrCa cell lines

To gain insight into the mechanism of ganetespib-induced cell death, we tested the effects of ganetespib on the cell cycle via flow cytometry. Representative intact (PCAP-1) and DGX-derived (PCAP-5) *Pten/Tp53* null mouse cell lines as well as the AR-dependent human LNCaP and LAPC4 cell lines were exposed to ganetespib (125 nM) for 24 hours, resulting in loss of S-phase and arrest in G2/M phase (Fig. 2E). In addition, PCAP cells demonstrated obvious sub-G1 peaks, characteristic of apoptotic populations. Various G2/M expressed proteins are clients of HSP90, including: CDK1, CHEK1, and CCNB1^17^. Exposure to ganetespib for 24 hours resulted in protein expression consistent with non-traditional G2/M arrest. There were significantly decreased levels of client proteins required for G2 cell cycle progression, including CDK1 in all cell lines and CCNB1 in three of the four cell lines. CCNB1 and CDK1 mRNA expression were also decreased (Supplementary Fig. 3A,B), suggesting multiple mechanisms are responsible for decreased protein levels and consistent with network drugs effecting multiple interrelated pathways. The HSP90 client CHEK1^34^, a critical sensor of DNA damage was also decreased following ganetespib treatment. In contrast, the protein expression of CCND1, which is not a direct HSP90 client, remained unchanged following ganetespib treatment (Supplementary Fig. 3C). These results demonstrate that ganetespib induced G2/M arrest and decreased protein expression of crucial G2/M checkpoint proteins required for progression.

### Ganetespib perturbs key signaling components of AR and PI3K/mTOR signaling

**Figure 3:**
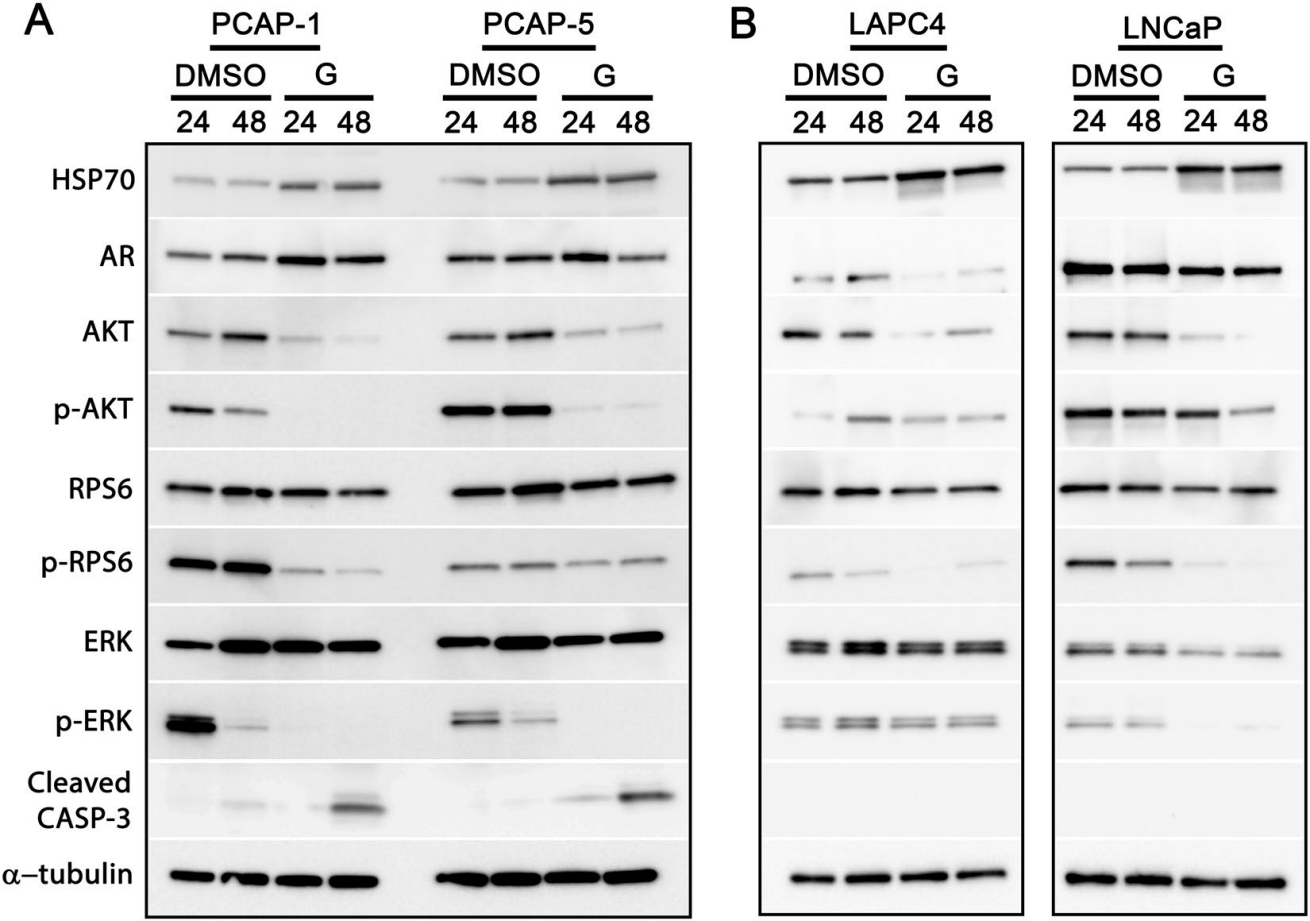
Ganetespib perturbs central PrCa signaling pathways in mouse and human cell lines. Western blots of extracts from cell lines as indicated were probed for signaling proteins. (**A**) PCAP-1 and PCAP-5 mouse cell lines were exposed to DMSO or 125 nM ganetespib (G) for 24 and 48 hours. (**B**) Human LAPC4 and LNCaP cells were treated with DMSO or 125 nM ganetespib (G) for 24 or 48 hours. Western blots are representative of three separate experiments and separate gels are outlined in black.

To begin mechanistically defining the target(s) of ganetespib activity, we analyzed underlying driver pathways of PrCa proliferation. Following exposure to ganetespib, phosphorylated levels of AKT, RPS6, and ERK exhibited decreases in nearly all mouse and human cell lines (Fig. 3A,B). Both total and phosphorylated levels of AKT, an HSP90 client, decreased, suggesting a direct effect of HSP90 inhibition on AKT stability. In addition, although RPS6 levels were stable, pRPS6 (serine 240/244) was decreased, consistent with inhibition of the upstream regulators, pAKT/mTORC1/S6K1. Similarly, for the mouse cell lines and LNCaP, upstream regulators of ERK phosphorylation appeared to be inhibited as p-ERK levels were reduced while total ERK expression was stable. We also observed induction of HSP70 protein expression, a known biomarker for HSP90 ATPase inhibition (Fig. 3A,B). Ganetespib led to decreased levels of AR total protein in LAPC4 and LNCaP cells, consistent with a previous report showing degradation of full-length AR in human PrCa cell lines^20^. By contrast, mouse AR mRNA and protein levels were slightly increased in PCAP cell lines following treatment with ganetespib *in vitro* (Fig. 3A and Supplementary Fig. 3D). Consistent with a pre-G1 population in the mouse cell lines (Fig. 2E), cleaved CASP3 was observed. In all, these data from multiple cell lines indicate that ganetespib-induced HSP90 inhibition causes multifactorial down-regulation of critical driver pathways involved in PrCa growth and survival including AR, AKT/RPS6, and ERK^26,35^. Importantly, ganetespib simultaneously decreased AR protein levels and inactivated p-AKT/RPS6 in human PrCa cells. These two pathways usually demonstrate opposite and compensatory levels of activation, which contribute to therapeutic resistance toward AR or PI3K pathway inhibition^26^.

### PB-Cre4 *Pten*^*fl/fl*^*;Tp53*^*fl/fl*^ GEMM progenitor cell proliferation and tumor progression are inhibited by ganetespib

**Figure 4:**
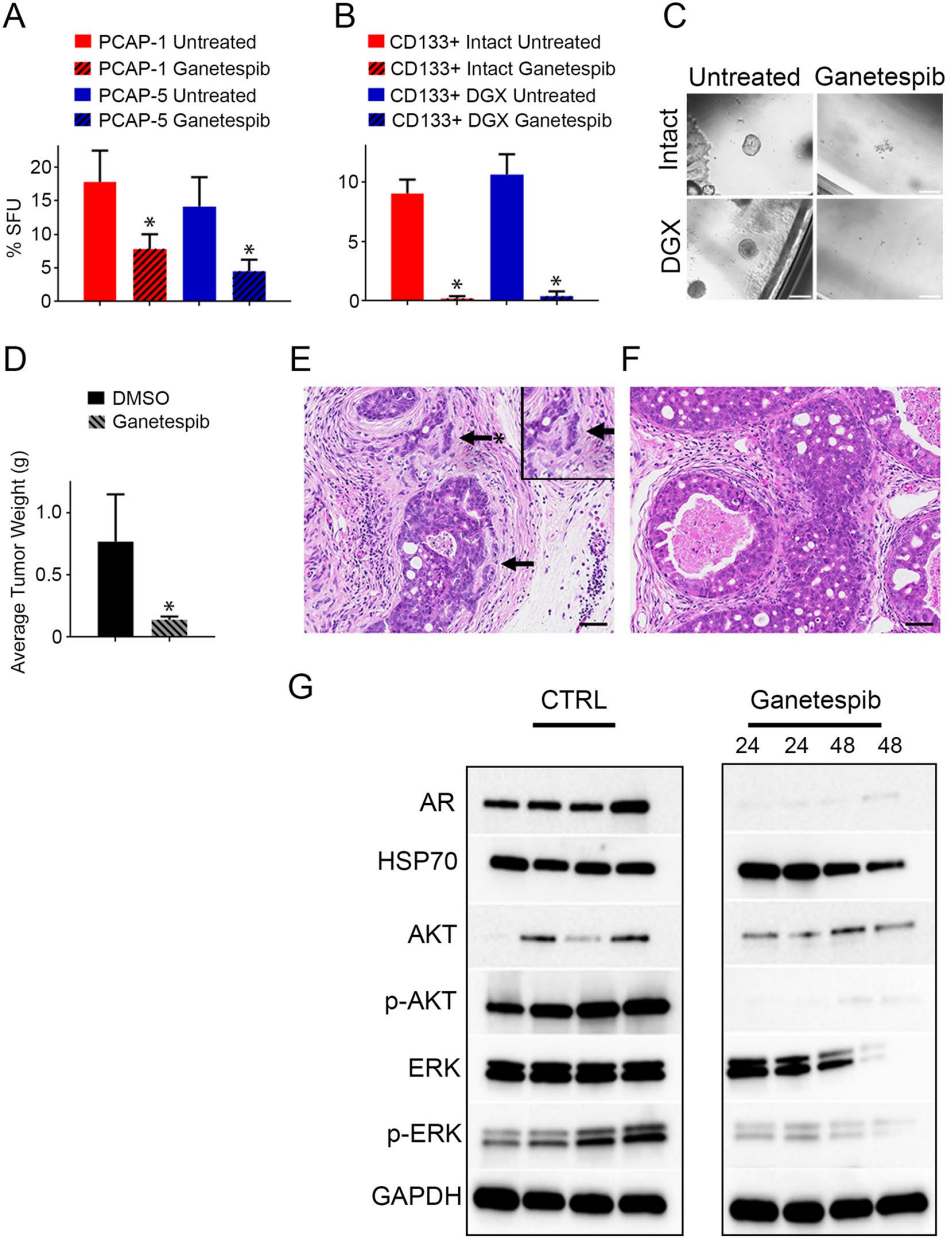
Ganetespib inhibits progenitor self-renewal and GEMM tumor progression. (**A**) PCAP cell lines embedded in Matrigel® were untreated or treated with 16 nM ganetespib for seven days and organoids > 50 μm were enumerated (n = 3, P < 0.05). (**B**) Primary tumor from intact or DGX-treated mice luminal (CD133^+^) organoids were untreated or treated *in vitro* with 16 nM ganetespib for seven days and sphere numbers determined (n = 3, P < 0.05). (**C**) Representative images of CD133^+^ organoid cultures following 7 days in the presence or absence of ganetespib (scale bars = 200 μm). (**D**) Tumor weights from PB-Cre4 *Pten*^*fl/fl*^*;Tp53*^*fl/fl*^ GEMM mice drugged intravenously with 125 mg/kg ganetespib (n = 6) or DMSO (n = 3) once a week for eight weeks. Data presented as average tumor weight + /− SEM (P < 0.05). (**E**,**F**) Representative photomicrographs of untreated (**E**) and ganetespib-treated (**F**) *Pten*^*fl/fl*^*;Tp53*^*fl/fl*^ GEMM mouse prostates (Hematoxylin and eosin stain, scale bar = 50 µm). Arrows identify stromal invasion. Asterisk indicates a magnified region of tissue. (**G**) Western blot analysis of individual PB-Cre4 *Pten*^*fl/fl*^*;Tp53*^*fl/fl*^ mouse tumor lysates either untreated or following 24 or 48 hours after intravenous ganetespib application. Control and treated samples were run on the same gel but cropped for convenient visualization.

Plastic and/or poorly differentiated progenitor cells are present in advanced PrCa^6,36^ and likely contribute to resistance toward current PrCa therapies^36,37^. Moreover, stem-like *PTEN*/*TP53* null PrCa cells are associated with a poor clinical prognosis^14^. To determine the effect of ganetespib on *Pten*/*Tp53* null PrCa progenitor cell self-renewal, we assayed sphere growth from individual cells in a 3-dimensional matrix. Following one week exposure to ganetespib, PCAP cell line sphere formation was significantly reduced (Fig. 4A). We further tested ganetespib efficacy on *Pten*/*Tp53* null PrCa stem/progenitor cells derived directly from primary tumors. Tumor cells were sorted to obtain the luminal (CD133+) fraction, which has been characterized to give rise to organoids initiated by bipotential and luminal stem/progenitor cells encompassing about 10 percent of the total tumor cells^15^. Organoids derived from PB-Cre4 *Pten*^*fl/fl*^*;Tp53*^*fl/fl*^ tumor-bearing intact and DGX-treated mice were incubated with the approximate *in vitro* IC50 of ganetespib (16 nM). After one week, there was almost no sphere formation observed in luminal organoids treated with ganetespib (Fig. 4B,C) compared to 10 percent in the untreated control organoids. These data show that ganetespib effectively inhibit primary *Pten*/*Tp53* null PrCa stem/progenitor cell self-renewal activity.

We assayed whether the efficacy of ganetespib extended to aggressive and invasive *in vivo PBCre4*;*Pten*^*fl/fl*^*;Tp53*^*fl/fl*^ tumors, which express intrinsic castration resistance^12^. Beginning at 12 weeks of age, when GEM mice typically display prostatic intraepithelial neoplasia (PIN) and often adenocarcinoma^24^, mice were treated with either DMSO or ganetespib for a total of 8 weeks. Mice treated with ganetespib presented with significantly decreased tumor weights relative to the control treatment group (Fig. 4D). In addition, pathological analysis showed stromal invasion by epithelia in most untreated mice (4 of 6) at 20 weeks of age (Fig. 4E, arrows), compared to mice treated with ganetespib that had almost no invasion (1/6) (Fig. 4F). These data suggest that ganetespib inhibits progression in established tumors. Lysates from four independent tumors, taken from ganetespib-treated mice, were compared to four tumors from untreated mice. Although there is considerable innate heterogeneity in tumor development in this model^24^ we observed reduced levels of AR, p-AKT, and p-ERK following ganetespib exposure. Although the mouse cell lines maintained AR levels in the presence of ganetespib (Fig. 3A), treatment of *PBCre4*;*Pten*^*fl/fl*^*;Tp53*^*fl/fl*^ mice demonstrated a clear loss of AR protein (Fig. 4G). In all, these data demonstrate that low nM ganetespib inhibits proliferation of luminal *Pten*/*Tp53* null PrCa stem/progenitor cells *ex vivo* and that ganetespib inhibits endogenous *Pten*/*Tp53* null prostate tumor progression *in vivo*.

### Ganetespib is efficacious against a clinically-representative panel of near-patient models

**Figure 5:**
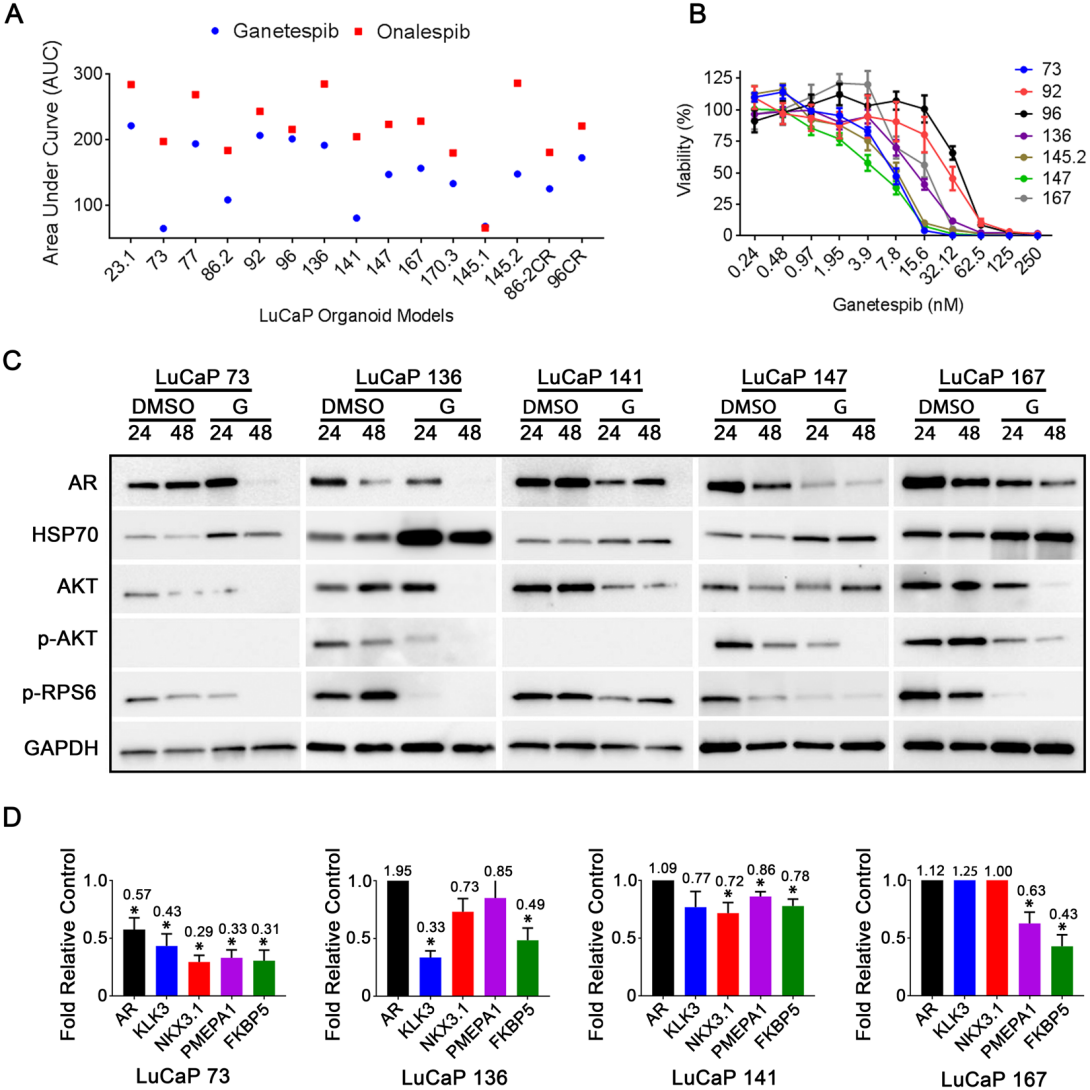
Ganetespib responses in a panel of LuCaP organoids. (**A**) HSP90 inhibitor data from 110 compound high-throughput screen of 15 LuCaP PDX-derived organoid models. Data presented as fitted area under curve (AUC) for 10 points between 30,000-1.52 nM. (**B**) Ganetespib dose response assays on LuCaP organoids. Data presented as percent viability +/− SEM. (**C**) Western blot biomarker analysis of indicated LuCaP organoids treated with DMSO or 125 nM ganetespib (G) for 24 or 48 hours. Images are representative of a minimum of two assays (n = 2) and vertical white space between images indicates separate gels. (**D**) qPCR analysis of AR target genes expression in several LuCaP organoid models following 24 hour exposure to 125 nM ganetespib. Data displayed as expression relative to untreated control +/− SEM (n = 3).

We sought to broadly analyze the activity of ganetespib in representative models of clinically advanced PrCa. Analyses of large PDX cohorts in several cancers have demonstrated high predictive value for genotypically-matched patient therapeutic responses^22^. The LuCaP PDX cohort are almost all derived from mCRPC and provide a platform for evaluating therapeutic responses relative to heterogeneous genotypic and phenotypic characteristics^38^. We have modified previously-described organoid culturing conditions to allow the *in vitro* growth of LuCaP PDX-derived tumor cells in three dimensional culture^23^ with maintenance of phenotypic characteristics, including AR signaling requirements. To investigate responsiveness across a panel of clinically-relevant genotypes and phenotypes we performed a screen on 15 PDX-derived LuCaP organoids using 110 drugs/compounds, ~60% of which are approved or in clinical trials. Two clinically-used HSP90 inhibitors, ganetespib and onalespib, were included and exhibited some of the broadest activity, with all the tested models demonstrating “responder” phenotypes (defined as IC50 < 1 μM and MAXR <20%) (Fig. 5A). The robust activity of HSP90 inhibitors for human CRPC PDX-derived organoids paralleled those observed in the MIPE screen (Fig. 1B). Ganetespib and onalespib displayed similar patterns of response across multiple models despite potency differences (Fig. 5A). Ganetespib showed sigmoidal dose response curves in validation assays of 3D organoid cultures (Fig. 5B), mirroring mouse and human PrCa cell lines (Fig. 2A–C). Ganetespib-responsive LuCaPs spanned a spectrum of CRPC phenotypes including: adenocarcinomas with wild-type AR (LuCaPs: 23.1, 73, 77, 96, 136, 147), adenocarcinomas with altered AR (LuCaPs: 86.2, 92, 141, 167), neuroendocrine disease (LuCaP 145.1, 145.2), and two experimentally-derived models (86.2CR, 96CR) selected as castrate-resistant in mouse hosts. Overall, the LuCaP organoid responses were not governed by obvious correlates of genotype or phenotype (Fig. 5A,B). The expression of cell signaling biomarkers was also consistent between models as the diverse LuCaP organoids treated with ganetespib displayed reductions in AR protein expression and PI3K signaling (i.e pAKT and/or pRPS6) (Fig. 5C). Consistent with reduced AR protein levels, AR target gene expression also was variably decreased across representative targets (Fig. 5D). A significant decrease in *FKBP5* was observed in each LuCaP tested, but the sensitivity of the other target genes was more variable. *PMEPA1* was decreased in three while *KLK3* and *NKX3*.*1* were decreased in two of four LuCaPs. In all, these results indicate ganetespib is efficacious for the heterogeneous CRPC landscape with no apparent intrinsic resistance, suggesting the potential for broad applicability in drug combination studies.

### Ganetespib inhibits PI3K signaling, decreases AR levels, and enhances the depth and durability of LuCaP 136 response to castration *in vivo*

**Figure 6:**
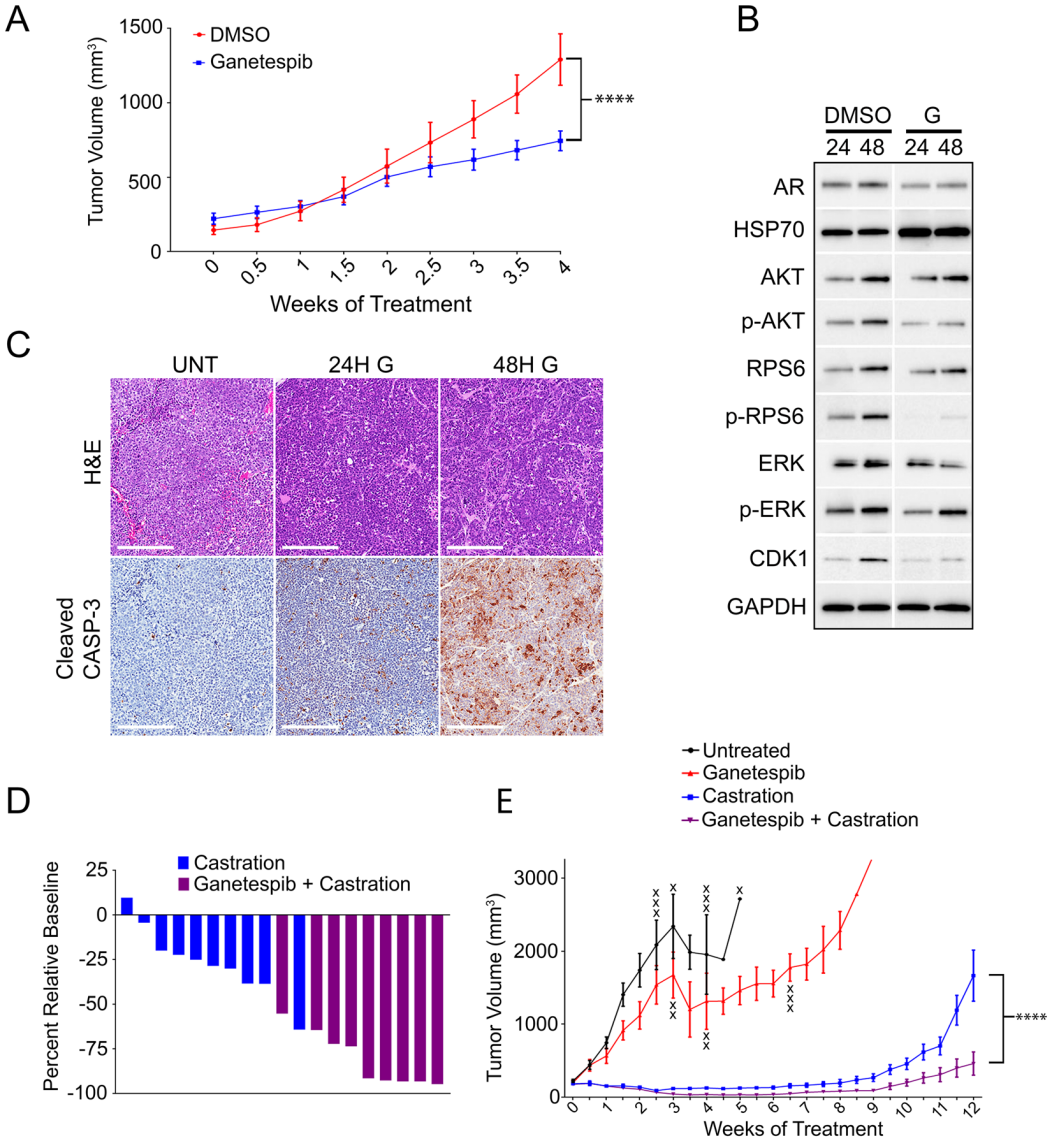
LuCaP 136 tumors treated with ganetespib have decreased PI3K and AR protein expression and show enhanced sensitivity to castration *in vivo*. (**A**) Tumor volume for LuCaP 136 treated with ganetespib (150 mg/kg, n = 8) or DMSO (n = 8) (P < 0.0005, two way anova with Bonferroni Corrections). (**B**) Western blots for the indicated biomarkers in individual tumors harvested 24 or 48 hours after ganetespib (G) treatment. Control and ganetespib treated samples were run on same gel but cropped during analysis for convenient visualization. (**C**) Representative H&E and cleaved caspase-3 IHC staining of LuCaP 136 tumors exposed to DMSO or 125 mg/kg ganetespib (G) for 24 or 48 hours (scale bars = 200 μm). (**D**) Waterfall plot showing individual tumor volumes at the greatest depth of ganetespib + castration PDX tumor response (4 week treatment) normalized to starting tumor volumes (Percent Relative Baseline). (**E**) LuCaP 136 tumor volumes for an untreated (n = 9) cohort or cohorts treated with castration (n = 10), ganetespib (125 mg/kg, n = 10), and ganetespib + castration (125 mg/kg, n = 9) (P < 0.005, 2-way anova with Bonferroni Corrections). All tumor measurements were averaged within respective cohorts and presented as averaged tumor volume +/− SEM. Data points are marked (X) to indicate the time at which individual mice were sacrificed after reaching the maximum allowed tumor size.

Ganetespib displayed broad efficacy across numerous clinically-representative organoid models. Single agent HSP90 inhibitors have lacked clinical efficacy^39^, but the potential of HSP90 inhibitors to potentiate drug activity and inhibit drug resistance mechanisms has spurred interest in HSP90 inhibitors for use in drug combinations^40^. To compare the previously characterized mouse *Pten/Tp53* null model, we used the LuCaP 136 PDX, which contains homozygous, loss of function aberrations in both *PTEN* and *TP53*^38^. We evaluated the efficacy of ganetespib against established, subcutaneous LuCaP 136 tumors and observed ~42% tumor growth inhibition (Fig. 6A). Exposure of LuCaP 136 tumor-bearing mice to ganetespib and biomarker analysis after 24 or 48 hours paralleled the *in vitro* biomarker changes including decreased AR, p-AKT, and p-RPS6 and increased HSP70 (Fig. 6B). In addition, immunohistochemical analysis of ganetespib treated tumors revealed active apoptosis observed as the presence of cleaved caspase-3 beginning at 24 hours post-treatment (Fig. 6C). These findings demonstrate the growth inhibitory activity of ganetespib for human *PTEN/TP53* altered CRPC, associated with pAKT and AR pathway biomarkers demonstrating reduced activity.

AR signaling blockade is almost always effective against metastatic PrCa initially, but the evolution of a variety of distinct resistance mechanisms, most of which directly or indirectly alter the AR pathway, lead to CRPC^1,2^. The ability of HSP90 inhibitors to target multiple pathways suggested ganetespib may work well in combination with AR signaling blockade to delay the development of resistance. To test this, we combined ganetespib with castration in mice bearing established LuCaP 136 tumors, which are castration-sensitive^38^ and responsive to ganetespib (Fig. 6A). Ganetespib deepened tumor response to castration with the most dramatic difference observed at 4 weeks relative to castration alone (Fig. 6D). This translated to a significant and sustained reduction in tumor volume in the ganetespib + castration cohort relative to other cohorts (Fig. 6E, Supplementary Fig. 4). The castration only cohort reached maximum allowable tumor burden prior to the ganetespib + castration cohort reaching even 50% of allowed burden (Fig. 6E). These *in vivo* findings in a PDX model representing a common CRPC genotype suggest that second-generation HSP90 inhibitors, like ganetespib, may enhance the response of AR blockade and delay the development of resistance.

## Discussion

Here we present evidence from multidrug screens and a variety of advanced prostate models that HSP90 inhibitors exhibit remarkably broad and potent activity. Using a high-throughput screening strategy directed against an intrinsically castration-resistant mouse model of *Pten/Tp53* null PrCa, we identified HSP90 inhibitors as potently anti-proliferative. The veracity of the MIPE screen for identifying aggressive PrCa vulernerabilities was validated by the identification of various anticipated pathway targets (e.g. PI3K/mTORC, EGFR, BRD4, and tubulin^35,41^) and has revealed additional targets of interest (see below). Similarly, in a screen of 15 LuCaP PDX-derived organoids against 110 drugs, which were selected for their potential as therapeutics in PrCa, we identified ganetespib and onalespib as two of the most broadly active agents at submicromolar concentrations. Although different LuCaP models vary as much as 10 fold in ganetespib IC50 values (Fig. 5B), >90% growth inhibition is broadly achieved below 100 nM. This is distinct from the description of HSP90 inhibitor non-responder cell lines in other cancers^42^, shows a lack of intrinsic resistance, and suggests a generalized high reliance in prostate cancer on HSP90 to buffer oncogenic stress. Thus, the potency and broad efficacy of ganetespib combined with a favorable pharmacological profile with minimal dose-limiting toxicity that prevented utility of first generation HSP90 inhibitors^33^ supports further in-depth analysis, particularly in combination studies (see below).

Decreased AR levels and AR-dependent signaling in various ganetespib-treated LuCaP adenocarcinoma models (Fig. 5) likely play a role in growth inhibition. However, the broad responsiveness of heterogeneous PrCa models here, including various AR-independent histologies, suggests multifactorial vulnerabilities and is consistent with over 200 HSP90 client proteins^17^. Following ganetespib exposure of the various PrCa models, we observed decreased levels of p-AKT and p-RPS6 (an mTORC1-S6K1 target), both *in vitro* and *in vivo*. PI3K is an important compensatory survival pathway for PrCa, and in particular, the simultaneous inhibition of AR and PI3K pathways has been shown to be a robust growth inhibitory combination^27^. In addition, our observation of ganetespib-mediated PBCre4;*Pten*^*fl/fl*^*;Tp53fl*^*/fl*^ mouse prostate tumor growth inhibition and correlative decreased pAKT levels is an example of additional AR independent network vulnerabilities. It has been proposed that high MYC levels are an important determinant of epichaperone formation and HSP90 inhibitor sensitivity^42^. However, we observed significant variability in MYC levels, including low amounts, which did not correlate with ganetespib response.

HSP90 inhibitors have had limited success as single agents in clinical trials of various solid tumors, perhaps due in part to adaptive stress responses^33^. Indeed, ganetespib was not efficacious in a small trial of CRPC patients who had failed multiple therapies^38^, although the failure of a single agent in highly advanced prostate cancer, which is characterized by genomic instability and multiple acquired resistance mechanisms^7^, is not unexpected. However, the ability of HSP90 inhibitors to target multiple signaling networks makes them an attractive class of drugs to use in combination therapies, both to amplify the efficacy of the companion drug and to inhibit potentially heterogeneous pathways contributing to the development of acquired resistance. Several HSP90 inhibitors remain in active clinical development for use in drug combinations^32,43^. To that end, we have investigated *in vivo* responses to ganetespib in combination with castration, ADT being the current standard of care for recurrent or metastatic PrCa. In combination studies using established *PTEN/TP53* null LuCaP 136 tumors, ganetespib added to castration compared to castration alone, led to deeper initial tumor regressions, and delayed progression to growth in a castrate environment (Fig. 6). These studies support continued investigations into optimizing various parameters, such as the timing of treatment and patient selection, for the use of second-generation HSP90 inhibitors in combination with androgen deprivation therapies. We anticipate that screening for additional efficacious combinations, including immunotherapy^44^, and the combined use of HSP90 inhibitors earlier in disease progression may further define the optimal context for clinical use of HSP90 inhibitors in CRPC.

In addition to identifying HSP90 inhibitors, the broad-based screen described here may be useful for future therapeutic targets of interest. Drugs targeting various pathways, which have potential relevance in human prostate cancer, were observed (Supplementary Table 3). We discuss a few pathways that were targeted by multiple different small molecules, increasing the likelihood of on-target screening specificity. For example, The NF-κB pathway has been described as functioning in advanced prostate cancer^45,46^, and multiple IKK2 and NF-κB inhibitors were active in the screen. Also, there is evidence for lipid oxidation in CRPC and a vulnerability for ferroptotic death^47^. The activity of PRDX (dioscin) and TRX/GPX (auranofin) inhibitors is consistent with sensitivity to elevated oxidative stress. In addition, several G2/M checkpoint inhibitors targeting ATR, CHEK1, or WEE1 demonstrated activity. Vulnerability to processes that lead to replication fork stress are a major source of G2/M checkpoint inhibitor sensitivity^48^. The vulnerability of the *Pten/Tp53* null cell lines used in the screen are particularly interesting considering that *PTEN* has been implicated in genomic stability functions including preventing premature mitotic entry, stabilizing stalled replication forks, and DNA decatenization^49,50^. Finally, drugs, such as the anti-malarial artemesin that were clinically developed for other indications and that may be usefully repurposed for the treatment of prostate cancer are representative of another category to be considered for further investigation. Overall, these screening results suggest utility in investigating the efficacy and extent of responsiveness in additional clinically-relevant models for several recently appreciated pathway/drug combinations.

## Materials and Methods

### Animal Care and Use

All *in vivo* experiments were completed at the NCI according to protocols approved by the NCI Animal Care and Use Committee.

### Cell Line Derivation from *PB-Cre4;Pten*^*fl/fl*^*;Tp53*^*fl/fl*^ GEMM

*PB-Cre4;Pten*^*fl/fl*^*;Tp53*^*fl/fl*^ mice were either untreated or treated with degarelix for two weeks. Degarelix treatment resulted in complete involution of seminal vesicles and, in the tumor, loss of nuclear AR and transient apoptosis^15^. Dissected prostate lobes were digested with Collagenase IV and DNase I. The resulting organoids were washed once with PBS and resuspended in Stemgent medium (CM-101,Cellaria, San Diego, CA), passed several times through a 19-gauge needle, and plated into a six-well Primaria plate (BD Biosciences, San Jose, CA). Eight cell lines derived from individual mice were established. The resultant lines were composed of morphologically uniform cells, determined to be absent PTEN and TP53, and designated PCAP 1–8 (Prostate Cancer Adenocarcinoma Pten/Tp53 null). PCAP cell lines were cultured in previously described conditions that promote the growth of primary breast luminal epithelium^30^ using serum -free Stemgent medium (CM-0100-A1, Cellaria) containing WIT-P (CM-101,Cellaria) supplement and 1% penicillin streptomycin. PCAP-4 was maintained in the described media supplemented with DHT.

### High-throughput MIPE Screen

Seven of the eight PCAP cell lines were processed to a single cell suspension by passing successively through 19, 21, 23, and 25 gage needles and filtered through a 40 μm nylon cell strainer (352340, Falcon). Each cell line was seeded at a density of 1,000 cells/well in 5 μL of media in a 1536 well plate and drugged (acoustically pinned) immediately post-plating encompassing 1912 compounds from the Mechanism Interrogation PlatE oncology library (MIPE)^51^. Plates were stored in an incubator and terminated using Cell Titer Glo 48 hours later. Luminescence readouts were processed through an informatics pipeline for curve response class fitting and classification^52^. Normalized data were sorted and analyzed for curve response class (efficacy) and MAXR (residual viability at the maximum concentration), with a preference for −1.1 and −1.2 curve class activity and MAXR values below 30% as described previously^53^.

### Three-dimensional culture of *ex vivo* organoids

Isolation, purification, and plating of *PB-Cre4;Pten*^*fl/fl*^*;Tp53*^*fl/fl*^ GEMM tumor cells to generate three-dimensional CD133^+^ and CD49^+^ mouse organoid cultures was done as described previously^15^. LuCaP PDX tumors were maintained, extracted, processed, and cultured as described^23^. Cells in a solution containing a 1:1 ratio of Matrigel^®^ Growth Factor Reduced (356231, BD Biosciences) and Advanced DMEM were seeded into 48-well (1.25 × 10^4^ cells per well) or 384-well (4 × 10^3^ cells per well) plates for dose response experiments. Dose response experiments on LuCaP organoids began 24 hours post-seeding and employed 11, two-fold serial dilutions starting with 250 nM as the maximum concentration. Media was exchanged every 3–4 days with or without ganetespib. Mouse and human organoid cultures incubated for a total of two weeks. Cell Titer Glo 3D (G9682, Promega) was used on all LuCaP organoids with the resultant luminescence normalized to DMSO control and averaged. All mouse and human organoid experiments were averaged from three independent experiments.

### High-throughput Organoid Screen

LuCaP PDX tumors were processed as described earlier and plated in a 384-well plate at a density of 4,000 cells per well in 20 μL comprised of 75% Matrigel^®^ Growth Factor Reduced (356231, BD Biosciences) and 25% Advanced DMEM. LuCaP-derived organoids were maintained in media described earlier and drugged the next day using ten, three-fold diluted concentrations on duplicate plates. Media was exchanged and plates were drugged 4 days later. The assay was terminated at day 7 using Cell Titer Glo 3D (G9683, Promega). Data were analyzed as described earlier.

### *In vivo* Efficacy Studies

Ganetespib powder (provided through an MCRADA with Synta Pharmaceuticals) was resupended in DMSO, dispensed dropwise into vehicle (20% Cremophor RH40, 80% Dextrose H_2_O), and solublized by 55° heat block incubation with intermittent 55° water bath sonication. GEMM: Twelve week old animals were treated with DMSO or 150 mg/kg ganetespib once weekly. At 20 weeks of age, mice were euthanized and dissected tumor weights were determined. LuCaP 136: Cells from processed frozen tumors were injected subcutaneously into cohorts of Athymic Nude-*Foxn1*^*nu*^ mice (Envigo Laboratories) or NOD *scid* gamma (NCI Fredrick). For the single agent experiment, intravenous drugging of the cohort was initiated simultaneously upon formation of established tumors (approximately 100 mm^3^) with 150 mg/kg ganetespib (n = 8) or DMSO (n = 8) once every seven days. For the combination study, cohorts were left untreated (n = 9), treated with 125 mg/kg ganetespib (n = 10), surgically castrated (n = 10), or surgically castrated and treated with ganetespib (n = 9) 48 hours later. Ganetespib treatment was initiated concurrently within the ganetespib alone and ganetespib + castration cohorts every seven days. Tumor volumes (4.188 × (Length/2) × (Width/2)^2^) were determined by caliper measurements made every 3–4 days. At the maximum allowable tumor burden (<2 cm^3^), mice were euthanized, and tumors were harvested. All mice were monitored for signs of toxicity such as weight loss or diarrhea. No toxic side effects were observed.

### Reagents

Antibodies and primers can be found in supplementary tables 1 and 2 respectively.

## Electronic supplementary material

Supplementary File

## Data Availability

The data generated and/or analyzed during the current study are available from the corresponding author on reasonable request.
