# Supplementary material for: High-throughput screens identify HSP90 inhibitors as potent therapeutics that target inter-related growth and survival pathways in advanced prostate cancer
Source: Sci Rep. 2018 Nov 22;8:17239. doi: 10.1038/s41598-018-35417-0 (PMC6250716; doi:10.1038/s41598-018-35417-0)
Supplement: Supplementary file 1 — Supplementary File [file 41598_2018_35417_MOESM1_ESM.docx]

**High-throughput screens identify HSP90 inhibitors as potent therapeutics that target inter-related growth and survival pathways in advanced prostate cancer**

Keith H. Jansson^1^, John B. Tucker^1^, Lauren E. Stahl^1^, John K. Simmons^2^, Caitlyn Fuller^1^, Michael L. Beshiri^1^, Supreet Agarwal^1^, Lei Fang^1^, Paul G. Hynes^1^, Aian Neil Alilin^1^, Ross Lake^1^, Yasmine C. Abbey^1^, Jacob Cawley^1^, Caitlin M. Tice^1^, JuanJuan Yin^1^, Crystal McKnight^3^, Carleen Klummp-Thomas^3^, Xiaohu Zhang^3^, Rajarshi Guha^3^, Shelley Hoover^2^, R. Mark Simpson^2^, Holly M. Nguyen^4^, Eva Corey^4^, Craig J. Thomas^3^, David A. Proia^5^, Kathleen Kelly^1^

**Supplementary Materials:**

**Materials and Methods**

**Supplementary Figure 1.** PCAP cell lines characterization.

**Supplementary Figure 2A.** Violin plot of ganetespib MAXR values from multiple cancer cell lines across various histologies analyzed in the MIPE screen at NCATS. **B.** Comparison of HSP70 protein induction between ganetespib and onalespib in PCAP-1 cells.

**Supplementary Figure 3.** qPCR and western blot analysis of G2/M progression proteins and AR in PCAP and LNCaP cell lines.

**Supplementary Figure 4.** Plot of individual tumor volumes for ganetespib + castration study on LuCaP 136 *in vivo*.

**Supplementary Table 1.** Antibodies used for western blots and IHC.

**Supplementary Table 2.** Primers used for Real Time qPCR.

**Supplementary Table 3.** Complete list of compounds from the MIPE library that displayed -1.1 or -1.2 curve response class activity in at least 4 of 7 PCAP cell lines.

**Supplementary Table 4.** List of compounds from the MIPE library that did not display activity against a majority (4 of 7) PCAP cell lines.

**Supplementary References.**

**Materials and Methods**

**Established Human Cell Line Culture Conditions:**

LAPC4, LNCaP, LNCaP-AR, DU145, and PC-3 cells were obtained from ATCC and cultured as described previously^1,2^.

**Efficacy Studies *in vitro*:**

PCAP cell lines were processed to single cells as described earlier. Processed cells were seeded in 96 well plates at a concentration of 2,000 cells/well to reach 40-50% confluency 24 hours post-plating. Ganetespib was purchased from Selleckchem.com (S1159) and the HSP90 inhibitors onalespib, SNX2112, and XL888 were generously provided by the laboratory of Dr. Len Neckers. The next day the seeded cells were drugged in a ten point, serial-diluted, dose response assay starting with 1μM as the maximum concentration. After a 48 hour incubation, the assay was terminated with MTS according to the manufacturer instructions (G3581, Promega) on a plate reader at 490nM absorbance. Background absorbance was removed and each well was normalized to the average of the DMSO treated wells. All concentrations of each drug were assayed in triplicate and each cell line was repeated a minimum of three times. Dose response data were plotted and analyzed statistically using GraphPad Prism 6 software.

**Flow Cytometry Analysis:**

PCAP, LNCaP, and LAPC4 cell lines at 50% confluency were drugged with DMSO vehicle control or 125nM ganetespib for 24 or 48 hours. Approximately five million live cells in 500uL PBS were fixed in 4.5 mL cold 100% EtOH, centrifuged at 1000 X g for 5 minutes, and resuspended in 1 mL of staining solution (0.1% Triton X-100, 0.2mg/mL RNAse A, 0.02mg/mL propidium iodide). A minimum of 30,000 events were measured for each cell line under each treatment condition on an LSR Fortessa (BD Biosciences) set to gate out aggregated cell doublets. Analysis was performed using FlowJo (FlowJo, LLC) and each experiment was repeated a minimum of 3 times.

**Three-Dimensional Culture of PCAP Cell Lines:**

PCAP cell lines were processed to single cells as described earlier. Fifty microliters of a solution containing a 1:1 ratio of Matrigel^®^ (354234, Corning) and 1000 cells in Stemgent was pipetted into each well of a 24-well plate, incubated for one hour at 37°, and 500uL of Stemgent was added to each well. Cultures were treated 24 hours post-plating with DMSO or 16nM ganetespib. Media was exchanged with or without ganetespib every 3-4 days for one week. Individual spheres were counted using phase contrast microscopy, with spheres defined as three dimensional organoids greater than 50μm. Each experiment was completed in duplicate a minimum of three times.

**Western Blot Analysis:**

All protein was extracted using RIPA buffer supplemented with phosphatase and protease inhibitors. Samples were prepared as follows. Cell lines: Cell lines were treated with DMSO or 125nM ganetespib 24 hours post-seeding (at 40-50% confluency) for 24 or 48 hours. Organoids: LuCaP organoids cultured for two weeks in 12-well plates were exposed to DMSO or 125nM ganetespib for 24 or 48 hours. Cells were extracted from the matrix by incubating 90 minutes with dispase (1 mg/mL). Tumors: Pb-Cre4+;Ptenfl/fl;P53fl/fl GEMM and LuCaP PDX tumors were harvested, snap frozen in liquid N_2_, and dounce homogenized. Western blots were performed on 4-20% Tris Glycine gels (05671093, BioRad), transferred to PVDF membranes, and blocked in PBS-T containing 5% dry milk. Primary antibodies **(Supplementary Table 1)** were incubated overnight at 4° C and secondary antibodies were applied the next day for 1 hour at room temperature using a concentration of 1:2000. Western blots were exposed using Clarity Western ECL Substrate (170-5061, BioRad) on a ChemiDoc Touch exposure system (BioRad). All western blots are representative of a minimum of three experiments. Control and ganetespib-treated samples were run adjacently on the same gels, unless indicated otherwise.

**Immunohistochemistry:**

Prostate tumors from *PB-Cre4;Pten^fl/fl^;Tp53^fl/fl^* GEMM mice and subcutaneous LuCaP 136 tumors were fixed in 4% PFA, sectioned, and H&E stained at Histoserv Inc (Germantown, MD). Slides were processed, stained, and imaged as described previously (**Supplementary** **Table 1**)^3^.

**Real-time qPCR:**

LuCaP 73, LuCaP 136, LuCaP 141, and LuCaP 167 tumors were processed and grown as described earlier. For organoid RNA, 500 μl of Trizol was dispensed into each well, incubated for 10 minutes, and extracted using 200 μL of a chloroform/isoamyl alcohol solution. RNA was purified with a Qiagen RNeasy Mini Kit (Qiagen cat. # 74106), cDNA was generated using using SuperScript VILO MasterMix (Invitrogen cat. #11755) on 100 ng of RNA followed by PCR utilizing a FastStart Universal SYBR Green Master Mix (Roche # 04913850001) done on an Applied Biosystems StepOnePlus Real-Time PCR machine. Primers are shown in Supplementary Table 2. Data were normalized to control employing the 2^-ΔΔC^_T_ method as described previously^4^.

**Statistical Analysis:**

Statistical analyses were performed using a two-tailed Student’s t-test, unless indicated otherwise. Asterisks mark comparisons considered statistically significant with a P-value<0.05, unless indicated otherwise.


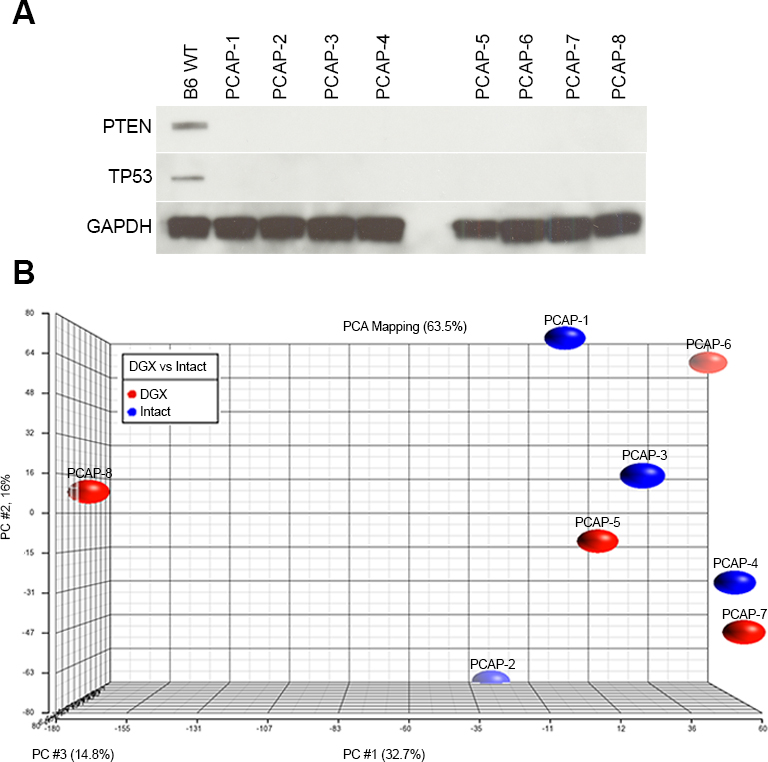


**Supplementary Figure 1.**

**Supplementary Figure 1.** PCAP cell lines characterization. **A.** Protein lysates from PCAP cell lines 1-8 were probed for PTEN, TP53, and GAPDH. **B.** Principle component analysis (PCA) of PCAP gene expression; color of oval represents castration status of the donor mouse.


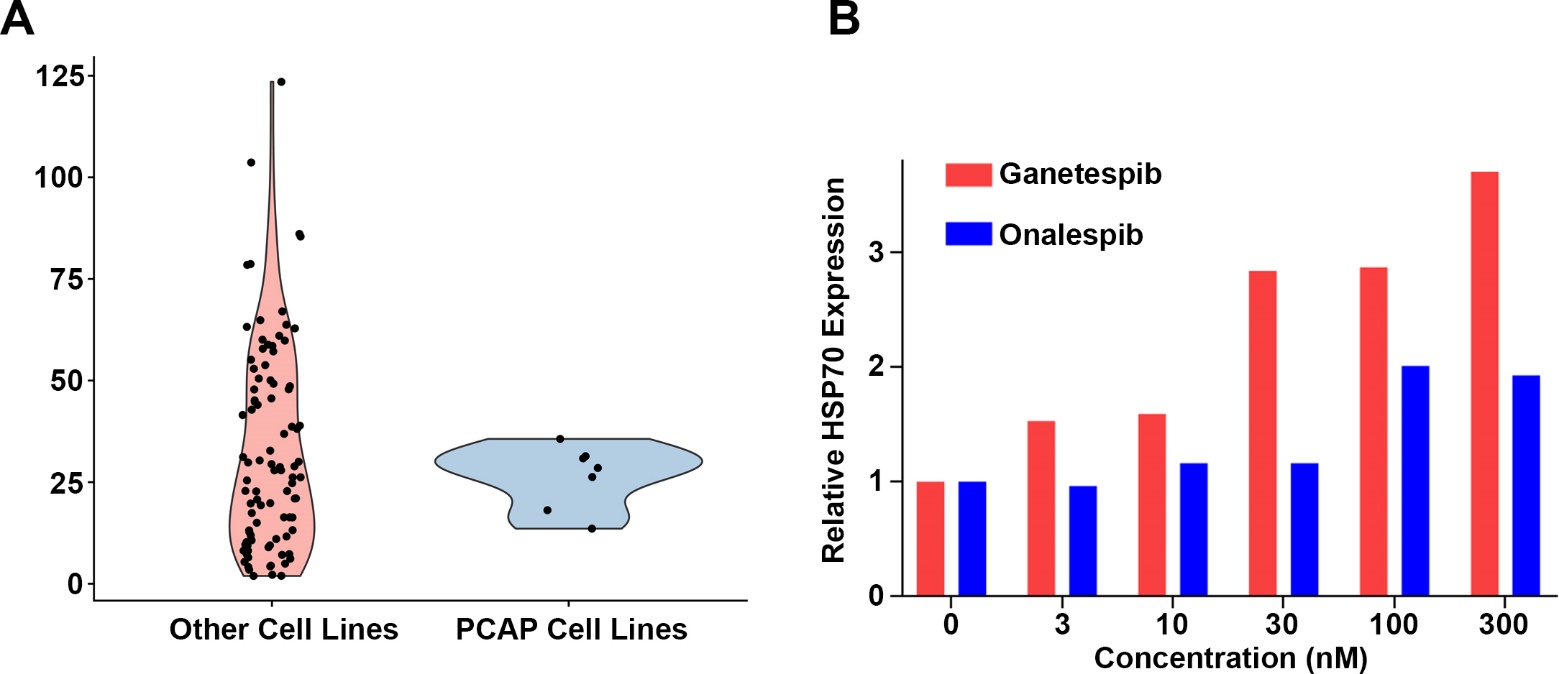


**Supplementary Figure 2.**

**Supplementary Figure 2A.** Violin plot of ganetespib MAXR values from multiple cancer cell lines across various histologies analyzed in the MIPE screen at NCATS. **B.** PCAP-1 cells were treated with indicated concentrations of ganetspib or onalespib for 24 hours and probed for HSP70 protein expression. Data were normalized to GAPDH for loading and then to HSP70 expression in the untreated cells.


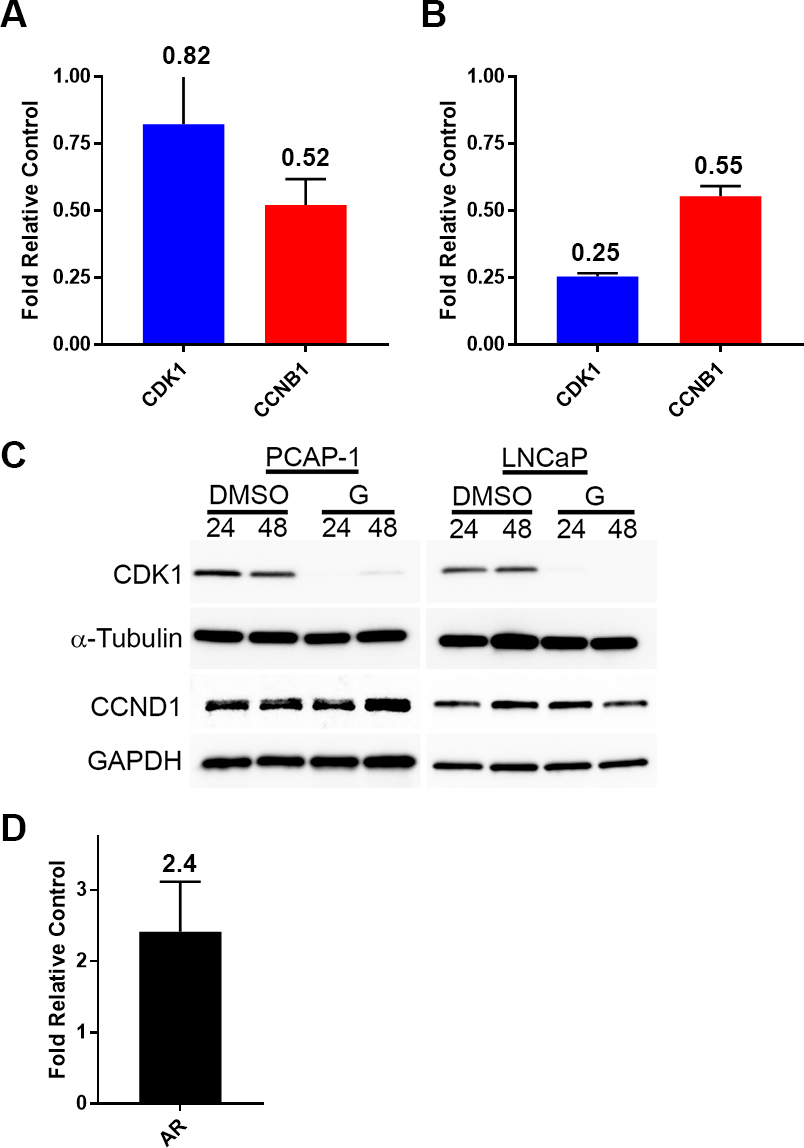


**Supplementary Figure 3.**

**Supplementary Figure 3. A.** qPCR analysis of PCAP-1 G2/M progression proteins following 24 hours 125 nM ganetespib treatment. **B.** qPCR analysis of LNCaP G2/M progression proteins after 24 hours 125 nM ganetespib exposure. **C.** PCAP-1 and LNCaP cells were treated with 125nM ganetespib for 24 or 48 hours and probed for CDK1 and CCND1. Vertical white space between images indicates separate gels. **D.** PCAP-1 *Ar* gene expression 24 hours after 125nM ganetespib treatment. qPCR data displayed as expression relative to untreated control +/- SEM (n=3).


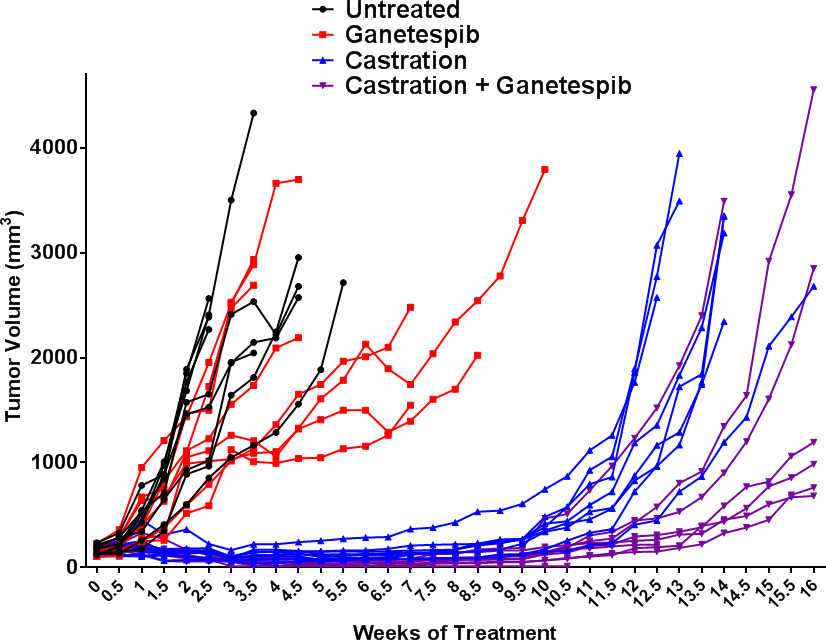


**Supplementary Figure 4.**

**Supplementary Figure 4.** Graph of individual LuCaP 136 tumors for untreated (black), ganetespib (red), castrated (blue), and ganetespib + castrated (purple) presented as tumor volume. Data plotted to 16 weeks post treatment initiation, when the last castrated mouse was taken after reaching max allowable tumor burden.

**Supplementary Table 1.** Antibodies used for western blots and IHC.

| **1° Antibody** | **Company, Catalogue #** | **Dilution** |
| --- | --- | --- |
| α tubulin | Sigma, T6199 | 1 : 2000 |
| AKT | Cell Signaling, 2938 | 1 : 1000 |
| p-AKT (S473) | Cell Signaling, 9271 | 1 : 1000 |
| AR | AbCam, ab133273 | 1 : 1000 |
| Caspase-3 | Cell Signaling, 9662 | 1 : 1000 |
| Cleaved Caspase-3 (IHC) | Cell Signaling, 9661 | 1 : 300 |
| CDC2 | Cell Signaling, 77055 | 1 : 1000 |
| CHEK1 | Santa Cruz, sc-8408 | 1 : 200 |
| CCNB1 | Cell Signaling, 4135 | 1 : 500 |
| CCND1 | Santa Cruz, sc-718 | 1 : 200 |
| ERK 1/2 | Cell Signaling, 4695, | 1 : 1000 |
| ERK 2 | Santa Cruz, sc-1647 | 1 : 500 |
| p-ERK 1/2 | Cell Signaling, 4370 | 1 : 1000 |
| HSP70 | Santa Cruz, sc-1060 | 1 : 1000 |
| PTEN | Cell Signaling, 9188S | 1 : 1000 |
| RPS6 | Cell Signaling, 2317S | 1 : 1000 |
| p-RPS6 (S240/244) | Cell Signaling, 22155 | 1 : 1000 |
| TP53 | Cell Signaling, 2524S | 1 : 1000 |

**Supplementary Table 2.** Primers used for Real Time qPCR.

| **Human** | **GENE** | **Forward (5'-3')** | **Reverse (5'-3')** |
| --- | --- | --- | --- |
|  | *AR* | GGAAGCTGCAAGGTCTTCTT | CGAAGACGACAAGATGGACAA |
|  | *CCNB1* | GGAAACATGAGAGCCATCCTAA | CTGCATGAACCGATCAATAATGG |
|  | *CDK1* | CACATGAGGTAGTAACACTCTGG | GTTCAGCAAATATGGTGCCTATAC |
|  | *FKBP5* | AAACGAAGGAGCAACAGTAGAA | TTGGAATGTCGTGGTCTTCTC |
|  | *HPRT1* | CTTTCCTTGGTCAGGCAGTATAA | AGTCTGGCTTATATCCAACACTTC |
|  | *KLK3* | CACACCCGCTCTACGATATG | GAGGTCCACACACTGAAGTT |
|  | *PMEPA1* | GTCTGCACGGTCCTTCATC | GATTCCGTTGCCTGACACT |
|  | *NKX3.1* | AGCCAGAAAGGCACTTGG | TCACCTGAGTGTGGGAGAA |
| **Mouse** | *Ar* | GTTGGCGGTCCTTCACTAAT | CTCATCCTCACACACTGGCT |
|  | *Ccnb1* | CAACTCTGCAGCACTACCTATC | CCACAGTTCACCATGACTACAT |
|  | *Cdk1* | AG GCGTTTGGAATACCGAT | AACCGGAGTGGAGTAACGAG |

**Supplementary Table 3.** Complete list of compounds from the MIPE library that displayed -1.1 or -1.2 curve response class activity in at least 4 of 7 PCAP cell lines. Sorted by mode of action.

| **Name** | **Target** | **Mode of Action (MoA)** |
| --- | --- | --- |
| Rolofylline | ADORA1 | Adenosine A1 antagonist |
| Cladribine | ADA | Adenosine deaminase inhibitor |
| A-674563 | AKT1 | Akt1/PKA inhibitor |
| Mithramycin | ADH1A | Alcohol dehydrogenase inhibitor |
| Temocapril hydrochloride | ACE | Angiotensin-I converting enzyme inhibitor |
| Spectinomycin hydrochloride |  | Antibiotic |
| Thiamphenicol |  | Antibiotic |
| Salinomycin | WNT1 | Anticoccidial/antibacterial |
| Antimycin A | ATP synthase | Antifungal agent |
| Natamycin | ergosterol | Antifungal agent |
| Artemisinin |  | Antimalarial agent |
| Artenimol |  | Antimalarial agent |
| Methylene blue |  | Antimalarial agent |
| Daunorubicin |  | Antineoplastic antibiotic |
| Echinomycin |  | Antineoplastic antibiotic |
| Elactocin |  | Antineoplastic antibiotic |
| Fenbendazole |  | Antiparasitic agent |
| Emetine |  | Antiprotozoal agent |
| ASR-isobudimer-SO2Ph-4-CH2OC(O)NMe2 |  | Artemesinin analogue |
| BTM-2C-dimer allyl oxime |  | Artemesinin analogue |
| BTM-2C-dimer ketone |  | Artemesinin analogue |
| Oligomycin A | ATP synthase | ATP synthase inhibitor |
| VE-821 | ATR | ATR kinase inhibitor |
| ABT-737 | BCL2L1 | Bcl-xL, Bcl-2, Bcl-w inhibitor |
| Navitoclax | BCL2L1 | Bcl-xL, Bcl-2, Bcl-w inhibitor |
| Carvedilol | ADRB1 | Beta1-adrenoceptor antagonist |
| I-BET762 | BRD4 | Bromodomain-containing protein 4 (Brd4, HUNK1) inhibitor |
| HMSL10077 | BTK | BTK inhibitor |
| Monatepil |  | Calcium channel blocker |
| Calhex-231 | CASR | Calcium-sensing receptor (CaSR) agonist |
| AT7519 | CDK1 | CDK 1 & 2 inhibitor |
| CGP-60474 | CDK1 | CDK1/2 inhibitor |
| NU-6027 | CDK1 | CDK1/2 inhibitor |
| RGB-286147 | CDK1 | CDK1/2/3/7/9 inhibitor |
| R-547 | CDK1 | CDK1/2/4 inhibitor |
| 7-Hydroxystaurosporine | CDK1 | CDK1/2/4/6 |
| Flavopiridol | CDK1 | CDK1/2/4/6/7/9 inhibitor |
| BS-194 | CDK1 | CDK1/2/5/9 inhibitor |
| Dinaciclib | CDK1 | CDK1/2/5/9 inhibitor |
| AZD-5438 | CDK1 | CDK1/Cyclin B1 inhibitor; CDK2/Cyclin A inhibitor; CDK2/Cyclin E inhibitor |
| SNS-032 | CDK2 | CDK2,7,9 inhibitor |
| PHA-690509 | CDK2 | CDK2/Cyclin A inhibitor |
| Fascaplysin | CDK4 | CDK4/6 inhibitor |
| DA-3003-1 | CDC25 | Cell division cycle CDC25 phosphatase inhibitor |
| Lumacaftor | CFTR | CFTR channel modulator |
| CHIR-124 | CHEK1 | Chk1 inhibitor |
| PF-477736 | CHEK1 | Chk1 inhibitor |
| Rabusertib | CHEK1 | Chk1 inhibitor |
| SCH-900776 | CHEK1 | Chk1 inhibitor |
| SM-164 | XIAP | cIAP1/2 and XIAP inhibitor |
| PD-173955 | SRC | cSRC inhibitor |
| Itraconazole |  | Cytochrome inhibitor |
| Methotrexate | DHFR | Dihydrofolate reductase (DHFR) inhibitor |
| Pralatrexate | DHFR | Dihydrofolate reductase (DHFR) inhibitor |
| Trimetrexate | DHFR | Dihydrofolate reductase (DHFR) inhibitor |
| 5-Azacitidine | DNMT1 | DNA methyltransferase (DNMT) inhibitor |
| NSC-48006 | DNMT1 | DNA methyltransferase (DNMT) inhibitor |
| Clofarabine |  | DNA polymerase inhibitor |
| Camptothecin | TOP1 | DNA topoisomerase I inhibitor |
| SN-38 | TOP1 | DNA topoisomerase I inhibitor |
| Aclarubicin | TOP2A | DNA topoisomerase II inhibitor |
| Doxorubicin | TOP2A | DNA topoisomerase II inhibitor |
| Epirubicin hydrochloride | TOP2A | DNA topoisomerase II inhibitor |
| Idarubicin hydrochloride | TOP2A | DNA topoisomerase II inhibitor |
| Mitoxantrone | TOP2A | DNA topoisomerase II inhibitor |
| Pirarubicin | TOP2A | DNA topoisomerase II inhibitor |
| KU 0060648 | PRKDC | DNA-dependent protein kinase (DNA-PK) inhibitor |
| Rifapentine | PRKDC | DNA-directed RNA polymerase inhibitor |
| Vanoxeamine |  | Dopamine reuptake inhibitor |
| Bupropion hydrochloride | SLC6A3 | Dopamine transporter (DAT) inhibitor |
| Pluripotin | RASAL1 | Dual RASGAP/Erk inhibitor |
| Afatinib | EGFR | EGFR (HER1; erbB1) inhibitor |
| AG-1478 | EGFR | EGFR (HER1; erbB1) inhibitor |
| Neratinib | EGFR | EGFR (HER1; erbB1) inhibitor |
| OSI-420 | EGFR | EGFR (HER1; erbB1) inhibitor |
| PD-153035 | EGFR | EGFR (HER1; erbB1) inhibitor |
| Pelitinib | EGFR | EGFR (HER1; erbB1) inhibitor |
| Fulvestrant | ESR1 | Estrogen receptor antagonist |
| HMSL10084 | FLT3 | FLT3 |
| GSK-1292263A | GPR119 | Glucose-dependent insulinotropic receptor (GDIR, GPR119) agonist |
| Cyanein | GBF1 | Golgi-specific brefeldin A-resistance guanine nucleotide exchange factor 1 (GBF1) inhibitor |
| 6-Bromoindirubin-3'-acetoxime | GSK3B | GSK-3 inhibitor |
| Alvespimycin hydrochloride | HSP90AB1 | Heat shock protein 90 (hsp90) inhibitor |
| AT-13387AU | HSP90AB1 | Heat shock protein 90 (hsp90) inhibitor |
| CNF-2024 | HSP90AB1 | Heat shock protein 90 (hsp90) inhibitor |
| Ganetespib | HSP90AB1 | Heat shock protein 90 (hsp90) inhibitor |
| KW-2478 | HSP90AB1 | Heat shock protein 90 (hsp90) inhibitor |
| Retaspimycin | HSP90AB1 | Heat shock protein 90 (hsp90) inhibitor |
| SNX-2112 | HSP90AB1 | Heat shock protein 90 (hsp90) inhibitor |
| SNX-5422 | HSP90AB1 | Heat shock protein 90 (hsp90) inhibitor |
| Tripterin | HSP90AB1 | Heat shock protein 90 (hsp90) inhibitor |
| VER-82576 | HSP90AB1 | Heat shock protein 90 (hsp90) inhibitor |
| Sapitinib | ERBB2 | HER2/3 (erbB2) inhibitor |
| Quisinostat hydrochloride | HDAC1 | Histone deacetylase (HDAC) 1 inhibitor |
| Trichostatin A | HDAC1 | Histone deacetylase (HDAC) 1 inhibitor |
| Mocetinostat | HDAC1 | Histone deacetylase (HDAC) 1 inhibitor |
| AR-42 | HDAC1 | Histone deacetylase (HDAC) 1/2 inhibitor |
| Pracinostat | HDAC1 | Histone deacetylase (HDAC) 1/2 inhibitor |
| ISOX | HDAC1 | Histone deacetylase (HDAC) 6 inhibitor |
| Abexinostat | HDAC1 | Histone deacetylase (HDAC) inhibitor |
| Dacinostat | HDAC1 | Histone deacetylase (HDAC) inhibitor |
| Givinostat hydrochloride | HDAC1 | Histone deacetylase (HDAC) inhibitor |
| M344 | HDAC1 | Histone deacetylase (HDAC) inhibitor |
| Panobinostat | HDAC1 | Histone deacetylase (HDAC) inhibitor |
| Romidepsin | HDAC1 | Histone deacetylase (HDAC) inhibitor |
| 2-Methoxyestradiol | HIF1A | Hypoxia inducible factor 1-alpha inhibitor |
| Birinapant | XIAP | IAP inhibitor |
| Phloretin | ICAM1 | ICAM1 and VCAM1 expression inhibitor |
| TPCA-1 | IKBKB | IKK beta inhibitor |
| GMX-1778 | IKBKB | IKK inhibitor |
| IMD-0354 | IKBKB | IKK-2 inhibitor |
| CAY10581 | IDO1 | Indoleamine 2,3-dioxygenase (IDO) inhibitor |
| AVN-944 | IMPDH1 | Inosine 5'-monophosphate dehydrogenase (IMPDH) inhibitor |
| Mycophenolate mofetil | IMPDH1 | Inosine 5'-monophosphate dehydrogenase (IMPDH) inhibitor |
| Mycophenolic acid | IMPDH1 | Inosine 5'-monophosphate dehydrogenase (IMPDH) inhibitor |
| NCGC00188382-01 | ITK | ITK inhibitor |
| NCGC00344990-01 | ITK | ITK inhibitor |
| NCGC00344999-01 | ITK | ITK inhibitor |
| Lestaurtinib | JAK1 | Jak/Tyk/Flt inhibitor |
| SR-3306 | MAPK8 | JNK 1/2/3 inhibitor |
| Posaconazole | CYP51A1 | Lanosterol 14alpha-demethylase inhibitor |
| AMG-47a | LCK | Lck kinase inhibitor |
| KHS101 |  | Lineage-specific differentiation enhancer |
| Azalomycin-B |  | Macrolide antibiotic |
| Pimecrolimus |  | Macrolide antibiotic |
| MI-2 | MALT1 | MALT1 inhibitor |
| AS-602801 | AS-602801 | MAPK8 |
| BI-D1870 | RPS6KA1 | MAPKAP-K1 (RSK; p90Rsk) inhibitor |
| Pimasertib | MAP2K1 | MEK 1/2 inhibitor |
| Trametinib | MAP2K1 | MEK 1/2 inhibitor |
| AZD-8330 | MAP2K1 | MEK inhibitor |
| KUC111774N-03 |  | Microphthalmia-associated transcription factor (MITF) inhibitor |
| Chlorhexidine | MMP1 | MMP inhibitor |
| GM-6001 | MMP1 | MMP inhibitor |
| BEZ-235 | MTOR | mTOR inhibitor |
| GDC-0980 | MTOR | mTOR inhibitor |
| PF-05212384 | MTOR | mTOR inhibitor |
| Torin-1 | MTOR | mTOR inhibitor |
| Torin-2 | MTOR | mTORC1 inhibitor |
| AZD-2014 | MTOR | mTORC1/2 inhibitor |
| AZD-8055 | MTOR | mTORC1/2 inhibitor |
| INK-128 | MTOR | mTORC1/2 inhibitor |
| KU-0063794 | MTOR | mTORC1/2 inhibitor |
| OSI-027 | MTOR | mTORC1/2 inhibitor |
| WYE-125132 | MTOR | mTORC1/2 inhibitor |
| Rotenone | NDUFAF1 | NADH-ubiquinone oxidoreductase (complex I) inhibitor |
| Withaferin A | NFKB1 | NF-kappaB Activation inhibitor |
| Bardoxolone methyl | NFKB1 | NF-kappaB signaling inhibitor |
| JTC-801 | OPRL1 | ORL1 (OP4, NOP) antagonist |
| Prasugrel | P2RY12 | P2Y12 (P2T) antagonist |
| KUC107871N-04 | p97 | p97 ATPase inhibitor |
| GB 83 | PAR2 | PAR2 antagonist |
| Amuvatinib | PDGFRA | PDGFR alpha inhibitor |
| Dioscin | PRDX1 | Peroxiredoxins (PRDX) 1/6 |
| MP-10 | PDE10A | Phosphodiesterase PDE10A inhibitor |
| Tadalafil | PDE5A | Phosphodiesterase V (PDE5) inhibitor |
| Verteporfin |  | Photosensitizer |
| CAY10505 | PIK3CG | PI3K gamma inhibitor |
| CUDC-907 | PIK3CA | PI3K inhibitor |
| GNE-477 | PIK3CA | PI3K inhibitor |
| GNE-493 | PIK3CA | PI3K inhibitor |
| PF-04691502 | PIK3CA | PI3K inhibitor |
| PIK-75 | PIK3CA | PI3K inhibitor |
| PKI-402 | PIK3CA | PI3K inhibitor |
| ZSTK-474 | PIK3CA | PI3K inhibitor |
| BAG-956 | PIK3CA | PI3K/PDK1 inhibitor |
| BKM-120 | PIK3CA | PI3Kalpha inhibitor |
| GSK-615 | PIK3CA | PI3Kalpha inhibitor |
| CH-5132799 | PIK3CA | PI3Kalpha, beta, gamma inhibitor |
| PI-103 | PIK3CA | PI3Kalpha, beta, gamma inhibitor |
| GSK-2126458 | PIK3CA | PI3Kalpha/beta/delta/gamma inhibitor |
| GDC-0349 | PIM3 | Pim-3 kinase inhibitor, mTOR |
| Triciribine | AKT1 | PKB/Akt inhibitor |
| Triciribine phosphate | AKT1 | PKB/Akt inhibitor |
| Midostaurin | PRKCA | PKC/flt3 inhibitor |
| Enzastaurin | PRKCA | PKCa, PKCb, PKCg inhibitor |
| ON-01910 | PLK1 | Polo-like kinase-1 (Plk-1) inhibitor |
| Monensin sodium salt |  | Polyether antibiotic |
| Nanchangmycin |  | Polyether antibiotic |
| Cantharidin | PPP1CA | PP-1 Inhibitor; PP-2A inhibitor |
| Mifepristone | PGR | Progesterone receptor antagonist |
| Bortezomib | PSMD1 | Proteasome inhibitor |
| Carfilzomib | PSMD1 | Proteasome inhibitor |
| Delanzomib | PSMD1 | Proteasome inhibitor |
| Ixazomib citrate | PSMD1 | Proteasome inhibitor |
| Marizomib | PSMD1 | Proteasome inhibitor |
| MG-115 | PSMD1 | Proteasome inhibitor |
| MG-132 | PSMD1 | Proteasome inhibitor |
| MLN-2238?? | PSMD1 | Proteasome inhibitor |
| Oprozomib | PSMD1 | Proteasome inhibitor |
| cycloheximide |  | Protein synthesis inhibitor |
| Berberine | PTP4A1 | Protein tyrosine phosphatase (PTP) inhibitor |
| Tioguanine |  | Purine antagonist |
| 2-Fluoroadenosine | PNP | Purine-nucleoside phosphorylase inhibitor |
| Carmofur |  | Pyrimidine antagonist |
| Floxuridine |  | Pyrimidine antagonist |
| Fluorouracil |  | Pyrimidine antagonist |
| MLN-2480 | BRAF | Raf kinase inhibitor |
| Resistomycin | POLR2A | RNA polymerase inhibitor |
| BI-78D3 | MAPK8 | SAPK1 (JNK) inhibitor |
| Idronoxil | SPHK1 | Sphingosine kinase 1 (SphK1) inhibitor |
| LLL-12 | STAT3 | STAT-3 inhibitor |
| Niclosamide | STAT3 | STAT-3 inhibitor |
| MK-8245 | SCD | Stearoyl-CoA desaturase inhibitor |
| Sepantronium bromide | BIRC5 | Survivin inhibitor |
| Fostamatinib disodium | SYK | Syk kinase inhibitor |
| SB-505124 |  | TGF-bR1 (ALK4/5) inhibitor |
| SB-525334 |  | TGF-bR1 (ALK5) inhibitor |
| Auranofin | TXNRD1 | Thioredoxin reductase inhibitor |
| Pemetrexed disodium | TYMS | Thymidylate synthase inhibitor |
| Raltitrexed | TYMS | Thymidylate synthase inhibitor |
| Trifluridine | TYMS | Thymidylate synthase inhibitor |
| CPG-52364 | TLR7 | TLR 7, 8 and 9 antagonist |
| Methylrosaniline chloride |  | TNFR1 (p55/CD120a) modulator |
| GSK-1016790A | TRPV4 | TRPV4 agonist |
| Lexibulin hydrochloride | TUBB | Tubulin depolymerization inhibitor |
| E-7010 | TUBB | Tubulin polymerization inhibitor |
| Indibulin | TUBB | Tubulin polymerization inhibitor |
| Ombrabulin | TUBB | Tubulin polymerization inhibitor |
| Vindesine sulfate | TUBB | Tubulin polymerization inhibitor |
| Vinflunine | TUBB | Tubulin polymerization inhibitor |
| Vinorelbine | TUBB | Tubulin polymerization inhibitor |
| Noscapine | TUBB | Tubulin polymerization inhibitor |
| Spautin-1 | USP10 | Ubiquitin specific peptidase (USP10/13) inhibitor |
| NCGC00262398 | USP2 | USP2 inhibitor |
| Vargatef | FLT1 | VEGFR-1/2/3 inhibitor |
| AG-13958 | FLT1 | VEGFR-2 (FLK-1/KDR) inhibitor |
| Takeda-6d | FLT1 | VEGFR-2 (FLK-1/KDR) inhibitor |
| MK-1775 | WEE1 | Wee1 kinase inhibitor |
| ICG-001 | WNT5A | Wnt signaling inhibitor |

**Supplementary Table 4.** List of compounds from the MIPE library that did not display activity against a majority (4 of 7) PCAP cell lines. Sorted by mode of action.

| **Name** | **Target** | **Mode of Action (MoA)** |
| --- | --- | --- |
| Aurora A Inhibitor I | AURKA | Aurora A inhibitor |
| MK-5108 | AURKA | Aurora A inhibitor |
| JNJ-7706621 | AURKA | Aurora kinase inhibitor |
| CCT-129202 | AURKA | Aurora kinase inhibitor |
| Hesperadin | AURKA | Aurora kinase inhibitor |
| PF-03814735 | AURKA | Aurora kinase inhibitor |
| ENMD-981693 | AURKA | Aurora-A inhibitor |
| Alisertib | AURKA | Aurora-A inhibitor |
| MLN-8054 | AURKA | Aurora-A inhibitor |
| ZM-447439 | AURKA | Aurora-A/B inhibitor |
| AZD-1152-HQPA | AURKA | Aurora-A/B inhibitor |
| AT-9283 | AURKA | Aurora-A/B inhibitor |
| TAK-901 | AURKA | Aurora-A/B inhibitor |
| PHA-680632 | AURKA | Aurora-A/B/C inhibitor |
| Tozasertib | AURKA | Aurora-A/B/C kinase inhibitor |
| SNS-314 | AURKA | Aurora-A/B/C kinase inhibitor |
| AMG-900 | AURKA | Aurora-A/B/C kinase inhibitor |
| Danusertib | AURKA | Aurora-A/B/C kinase inhibitor |
| CYC-116 | AURKA | Aurora-A/B/C kinase inhibitor |
| CCT-137690 | AURKA | Aurora-A/B/C kinase inhibitor |
| GSK-1070916A | AURKB | Aurora-B/C inhibitor |
| Abiraterone | AR | Androgen biosynthesis inhibitor |
| Orteronel |  | Androgen biosynthesis inhibitor |
| Andarine | AR | Selective androgen receptor modulators (SARM) |
| Ostarine | AR | Selective androgen receptor modulators (SARM) |
| Galeterone | AR | Selective androgen receptor modulators (SARM) |
| Flutamide | AR | Androgen receptor antagonist |
| Nilutamide | AR | Androgen receptor antagonist |
| Hydroxyflutamide | AR | Androgen receptor antagonist |
| Cyproterone acetate | AR | Androgen receptor antagonist |
| Finasteride | AR | Androgen receptor antagonist |
| Diindolylmethane | AR | Androgen receptor antagonist |
| (R)-Bicalutamide | AR | Androgen receptor antagonist |
| Bicalutamide | AR | Androgen receptor antagonist |
| MDV-3100 | AR | Androgen receptor antagonist |
| RD-162 | AR | Androgen receptor antagonist |
| 17 alpha-propionate | AR | Androgen receptor antagonist |
| ARN-509 | AR | Androgen receptor antagonist |
| INO-1001 | PARP1 | PARP inhibitor |
| AG-14361 | PARP1 | PARP-1 inhibitor |
| Iniparib | PARP1 | PARP-1 inhibitor |
| A-966492 | PARP1 | PARP-1 inhibitor |
| Olaparib | PARP1 | PARP-1/PARP-2 inhibitor |
| Veliparib | PARP1 | PARP-1/PARP-2 inhibitor |
| Rucaparib | PARP1 | PARP-1/PARP-2 inhibitor |
| Niraparib | PARP1 | PARP-1/PARP-2 inhibitor |
| IWP-2 |  | Porcupine (Wnt) inhibitor |
| LGK974 |  | Porcupine (Wnt) inhibitor |
| PNU-74654 | WNT5A | Wnt signaling inhibitor |
| KY02111 | WNT5A | Wnt signaling inhibitor |
| Wnt-C59 | WNT5A | Wnt signaling modulator |
| Salinomycin | WNT1 | Anticoccidial/Antibacterial |

**Supplementary References**

1 Yin, J. *et al.* Activation of the RalGEF/Ral pathway promotes prostate cancer metastasis to bone. *Mol Cell Biol* **27**, 7538-7550, doi:10.1128/MCB.00955-07 (2007).

2 Klein, K. A. *et al.* Progression of metastatic human prostate cancer to androgen independence in immunodeficient SCID mice. *Nat Med* **3**, 402-408 (1997).

3 Abou-Kheir, W. G., Hynes, P. G., Martin, P. L., Pierce, R. & Kelly, K. Characterizing the contribution of stem/progenitor cells to tumorigenesis in the Pten-/-TP53-/- prostate cancer model. *Stem Cells* **28**, 2129-2140, doi:10.1002/stem.538 (2010).

4 Livak, K. J. & Schmittgen, T. D. Analysis of relative gene expression data using real-time quantitative PCR and the 2(-Delta Delta C(T)) Method. *Methods* **25**, 402-408, doi:10.1006/meth.2001.1262 (2001).
